# Developmental fidelity is imposed by genetically separable RalGEF activities that mediate opposing signals

**DOI:** 10.1371/journal.pgen.1008056

**Published:** 2019-05-14

**Authors:** Hanna Shin, Christian Braendle, Kimberly B. Monahan, Rebecca E. W. Kaplan, Tanya P. Zand, Francisca Sefakor Mote, Eldon C. Peters, David J. Reiner

**Affiliations:** 1 Institute of Biosciences and Technology, Texas A&M Health Science Center, Texas A&M University, Houston, TX, United States of America; 2 Université Côte d’Azur, CNRS, Inserm, IBV, Nice, France; 3 Lineberger Comprehensive Cancer Center, University of North Carolina, Chapel Hill, NC, United States of America; 4 Department of Pharmacology, University of North Carolina, Chapel Hill, NC, United States of America; Brown University, UNITED STATES

## Abstract

The six *C*. *elegans* vulval precursor cells (VPCs) are induced to form the 3°-3°-2°-1°-2°-3° pattern of cell fates with high fidelity. In response to EGF signal, the LET-60/Ras-LIN-45/Raf-MEK-2/MEK-MPK-1/ERK canonical MAP kinase cascade is necessary to induce 1° fate and synthesis of DSL ligands for the lateral Notch signal. In turn, LIN-12/Notch receptor is necessary to induce neighboring cells to become 2°. We previously showed that, in response to graded EGF signal, the modulatory LET-60/Ras-RGL-1/RalGEF-RAL-1/Ral signal promotes 2° fate in support of LIN-12. In this study, we identify two key differences between RGL-1 and RAL-1. First, deletion of RGL-1 confers no overt developmental defects, while previous studies showed RAL-1 to be essential for viability and fertility. From this observation, we hypothesize that the essential functions of RAL-1 are independent of upstream activation. Second, RGL-1 plays opposing and genetically separable roles in VPC fate patterning. RGL-1 promotes 2° fate via canonical GEF-dependent activation of RAL-1. Conversely, RGL-1 promotes 1° fate via a non-canonical GEF-independent activity. Our genetic epistasis experiments are consistent with RGL-1 functioning in the modulatory 1°-promoting AGE-1/PI3-Kinase-PDK-1-AKT-1 cascade. Additionally, animals lacking RGL-1 experience 15-fold higher rates of VPC patterning errors compared to the wild type. Yet VPC patterning in RGL-1 deletion mutants is not more sensitive to environmental perturbations. We propose that RGL-1 functions to orchestrate opposing 1°- and 2°-promoting modulatory cascades to decrease developmental stochasticity. We speculate that such switches are broadly conserved but mostly masked by paralog redundancy or essential functions.

## Author summary

Developmental signals are increasingly conceptualized in the context of networks rather than linear pathways. Patterning of *C*. *elegans* vulval fates is mostly governed by two major signaling cascades that operate antagonistically to induce two cell identities. An additional pair of minor cascades support each of the major cascades. All components in this system are conserved in mammalian oncogenic signaling networks. We find that RGL-1, a component of one of the minor cascades, performs two antagonistic functions. Its deletion appears to abolish both opposing modulatory signals, resulting in a 15-fold increase in the basal error rate in development of these cells. We hypothesize that the bifunctional RGL-1 protein defines a novel mechanism by which signaling networks are interwoven to mitigate developmental errors.

## Introduction

**Fig 1:**
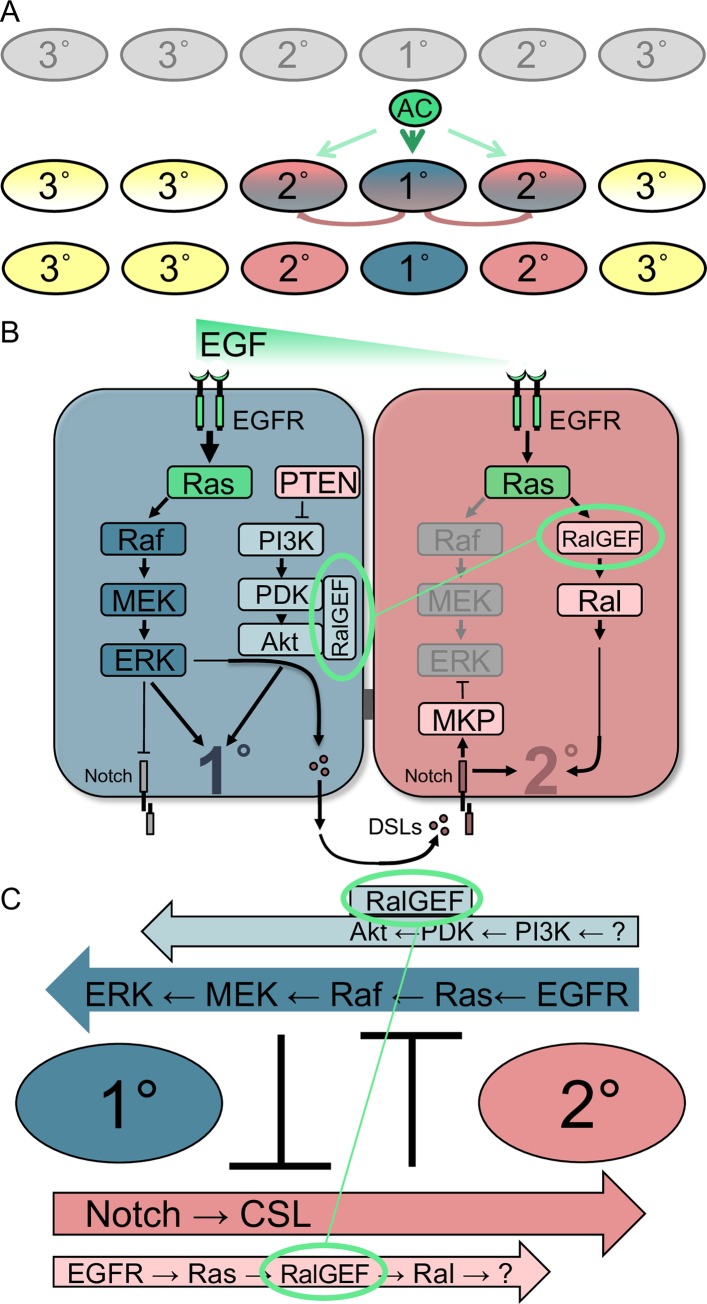
Schematics of VPC fate patterning and its signaling network. **A)** Initially equipotent VPCs are induced by the Anchor Cell (AC) to assume the 3°-3°-2°-1°-2°-3° pattern of fates (anterior-to-posterior), based on their position relative to the AC. Over time, induced VPCs progress from naïve to initially specified to terminally committed to their fates, represented by equipotent and uninduced (gray) progressing through initially specified (hybrid colors with one color dominant) to terminally committed to 1° (blue), 2° (rose) or 3° (yellow) fates. Yet the precise time course, molecular steps and network re-wiring events required for this progression are still unclear. **B)** The synthesis of the Sequential Induction, Graded Signal, and Mutual Antagonism models of the VPC patterning signal transduction network. The hypothesized dual functions of RGL-1/RalGEF are superimposed in green. Names of human protein orthologs are used for ease of understanding between diverse experimental systems: EGF = LIN-3, EGFR = LET-23, Ras = LET-60, Raf = LIN-45, MEK = MEK-2, ERK = MPK-1, DSLs = DSL ligands, Notch = LIN-12, RalGEF = RGL-1, Ral = RAL-1, MKP (MAP kinase/ERK phosphatase) = LIP-1, PTEN = DAF-18, PI3K = AGE-1, PDK = PDK-1, Akt = AKT-1. Necessary and sufficient cascades are in dark colors (dark blue for 1°-promoting LET-23/EGFR-LET-60/Ras-LIN-45/Raf-MEK-2/MEK-MPK-1/ERK; dark rose for LIN-12/Notch and the CSL transcriptional complex, CSL not pictured). Modulatory cascades are shown in lighter colors (light blue for 1°-promoting AGE-1/PI3K-PDK-1/PDK-AKT-1/Akt, with light rose for inhibitory DAF-18/PTEN lipid phosphatase; light rose for 2°-promoting RGL-1/RalGEF-RAL-1/Ral). Green represents proteins capable of promoting 1° or 2° fate, like LIN-3/EGF, LET-23/EGFR and LET-60/Ras, depending on signal dose and as yet unknown factors. Mutual antagonism operates by excluding potentially contradictory signals from initially specified VPCs: in presumptive 1° cells, LIN-12/Notch is internalized and degraded (gray), while in presumptive 2° cells MPK-1/ERK activation is repressed by transcriptional activation of LIP-1/MKP/DUSP/Erk phosphatase (and down-regulation of ral-1 promoter activity, not illustrated; [10]). The putative RGL-1/RalGEF Balanced Switch is circled in green both in presumptive 1° and presumptive 2° cells, and connected by a thin green line to represent the hypothetical switch in signaling activity. **C)** A wiring diagram of the naive 1°/2° VPC patterning signaling network, illustrating parallel and anti-parallel signals, with essential signals in dark blue and rose, and modulatory signals in light blue and rose. Data support RGL-1/RalGEF functioning in antagonistic 1°-promoting (non-canonical) and 2°-promoting (canonical) cascades. Deletion of *rgl-1* would perturb both modulatory cascades but not alter the balance of 1°- and 2°-promoting signals, even in sensitized backgrounds.

Developmental patterning of the *C*. *elegans* vulva precursor cell (VPC) fates is a textbook system for analysis of cell-cell signaling. The vulva develops from six roughly equipotent VPCs–P3.p through P8.p –that are induced to assume a 3°-3°-2°-1°-2°-3° pattern of cells fates. The anchor cell (AC) in the somatic gonad produces the LIN-3/EGF inductive signal (Fig 1A). LIN-3 signals via the LET-23/EGFR-LET-60/Ras-LIN-45/Raf-MEK-2/MEK-MPK-1/ERK canonical MAP kinase cascade to induce 1° fate (Fig 1B; [1]). In turn, redundant DSL ligands produced by presumptive 1° cells induce neighboring VPCs via LIN-12/Notch to become 2° [2, 3]; LIN-12 is necessary and sufficient for 2° fate [4] and controls 2°-specific transcriptional targets (Fig 1B; [5–7]). A graded signal was also found to contribute to 2° fate induction [8–10], but a mechanism by which this signal is mediated was lacking.

We identified the mechanism by which the 2°-promoting activity of the graded EGF signal was transduced. To interpret different doses of EGF signal, LET-60/Ras dynamically switches effectors during VPC fate patterning, from LIN-45/Raf signaling through the canonical MEK-2/MEK-MPK-1/ERK MAP kinase cascade to promote 1° fate, to RGL-1/RalGEF-RAL-1/Ral signaling to promote 2° fate [10, 11]. We further found that RAL-1 promotes 2° fate through EXOC-8/Exo84, a subunit of the heterooctameric exocyst complex, and a GCK-2/MAP4 kinase-PMK-1/p38 MAP kinase cascade [12].

Ras is the most mutated mammalian oncoprotein: more than a quarter of all tumors harbor activating mutations in Ras [13]. Three main oncogenic Ras effector cascades have been identified (S1 Fig). Two of these, the Raf-MEK-ERK MAP kinase and the PI3-Kinase-PDK-Akt cascades, have been extensively studied and pharmacologically targeted [14–16]. The RalGEF-Ral effector signal is poorly characterized, though it may be as important for oncogenesis as the Raf and PI3K cascades [17–22]. Ras binds and activates RalGEF, an exchange factor that stimulates GTP loading on the Ral small GTPase. Ral is a Ras-like small GTPase subfamily, in the Ras family of the Ras superfamily, and is conserved throughout Metazoa [20]. For most Ras family members, including Ral, this GDP/GTP cycle is controlled by activating GEFs and inactivating GAPs. Despite their structural and primary sequence similarities, Ral interacts with a very different set of effectors than does Ras [23].

All three oncogenic Ras effectors are involved as essential or modulatory cascades promoting 1° or 2° VPC fate in *C*. *elegans*. The essential LET-60/Ras-LIN-45/Raf-MEK/MEK-2-ERK/MPK-1 MAP kinase cascade promotes 1° fate with support of the modulatory AGE-1-PDK-1-AKT-1 cascade [24], while the essential LIN-12/Notch cascade promotes 2° fate with support of the modulatory LET-60/Ras-RGL-1-RAL-1 cascade.

Here we pursue the unexpected findings that RGL-1/RalGEF is functionally non-equivalent to RAL-1 in two distinct ways. First, while RAL-1 is essential for viability and fertility [10, 25], RGL-1 is inessential. Second, RGL-1 and RAL-1 are non-equivalent in VPC fate patterning. While RAL-1 functions as a simple intermediary to propagate the 2°-promoting LET-60-RGL-1-RAL-1 signal, RGL-1 additionally performs an opposing, putative 1°-promoting function that offsets its canonical 2°-promoting function. As a consequence, deletion of RGL-1 has no net effect on the delicate balance of 1°- and 2°-promoting signals in sensitized mutant backgrounds. Using GEF-specific mutations and genetic bypass experiments, we show that the opposing functions of RGL-1 in VPC fate patterning are genetically separable. In the context of mammalian studies that argue that RalGEF physically interacts with PDK and Akt as a scaffold [26, 27], our genetic epistasis results are consistent with RGL-1 functioning as a scaffold for PDK-1 and AKT-1 in the modulatory 1°-promoting AGE-1/PI3K cascade. Our analysis raises the question of how activity in two apparently opposing cascades contribute to VPC fate patterning. Comparing VPC fate patterning in different environments, we found that the error rate in patterning was 15-fold higher in *rgl-1* deletion mutants than in the wild type. We hypothesize that the two opposing activities of RGL-1, which tie together the two opposing 1°- and 2°-promoting modulatory cascades, are orchestrated to reduce the level of noise in the signaling network, and hence reduce the rate of ambiguous fates or mis-patterning events.

## Results

The impetus for this analysis was two enigmatic genetic observations. First, deletion of *ral-1* but not *rgl-1* confers severe deficits in growth, cell polarity, and fertility. Second, deletion of RAL-1 but not RGL-1 confers a net effect on 1° vs. 2° VPC induction. Thus, despite the linear Ras-RalGEF-Ral signaling module defined in mammals and validated in *C*. *elegans* VPC fate patterning, in two different ways RGL-1 is non-equivalent to RAL-1. By pursuing these two genetic observations, we arrived at important models regarding the multiple roles of RalGEF and Ral in signal transduction and development.

### The *C*. *elegans* RalGEF ortholog, RGL-1, is non-essential

*ral-1* is an essential gene. *ral-1(tm2760)* deletes half of the G-T dinucleotide of the intron 2 splice donor site, and thus likely a strong loss-of-function allele [10]. *ral-1(tm5205)* deletes exons 2 and 3 [25], which encode extensive RAL-1 sequences conserved throughout Metazoa and required for small GTPase function, and is therefore null.

We previously described that *ral-1(tm2760)* animals are sterile but otherwise wild type. Efforts to feed or inject dsRNA in the RNAi hypersensitive *rrf-3* background failed to phenocopy this sterility. Yet consistent with our depletion by bacterially mediated RNAi and injected dsRNA, *tm2760* abrogated the 2°-promoting activity of RAL-1 [10].

Subsequent analysis of *ral-1(tm5205)* and a *ral-1(M*^*-*^*Z*^*-*^*)* construct led to the conclusion that RAL-1 function is maternally rescued and necessary for various facets of embryonic, post-embryonic, and germline development that require function of the PAR complex in cell polarity [25]. This function is ascribed to a central role of RAL-1 in the exocyst complex, as described for mammals [28–34]. No phenotypes have been ascribed to LET-60/Ras that could be associated with exocyst and PAR complex function [35]. We observed that *ral-1(tm5205)* larvae from a heterozygous mother are sickly in the fourth larval stage and become sterile adults, but VPCs in an otherwise wild-type background are patterned normally (N = 52).

Mammalian studies suggest that Ral associates with the exocyst in an activity-dependent manner to promote complex assembly [28–34]. Thus, abrogation of GTP-loading, either through disruption of RalGEF activity or Ral GTP-loading activity, should phenocopy loss of Ral and result in defective exocyst function. Since *C*. *elegans* encodes only a single RalGEF ortholog [10], we tested whether the RGL-1/RalGEF is required for all described RAL-1 activities.

Strikingly, four different deletion mutants of RGL-1 are superficially wild type, develop normally, are fertile, and can be grown indefinitely in culture (Table 1). One of these mutations, *fax-1(gm27*), deletes several neighboring genes on the X chromosome, including *rgl-1* [36]. We developed robust primer sets to detect *ok1921* and *tm2255* (see Methods; S6 Fig, S7 Fig). These primers failed to amplify *rgl-1* sequences from the *gm27* mutant animal. Mutants for the heterodimeric RalGAP we also healthy and fertile [37].The observation that deletion of RGL-1/RalGEF does not result in the same sickly/sterile and maternal affect lethality phenotypes as deletion of RAL-1 raises the interesting possibility that Ral and RalGEF are functionally non-equivalent, contrary to the model derived from biochemical experiments in mammalian cell culture.

**Table 1:** *rgl-1* and *ral-1* alleles and mutant phenotypes.

Allele	Lesion	Superficial Phenotype	Net VPC alteration^a^
*rgl-1(ok1921)*	In-frame deletion, GEF domain	Wild type	none
*rgl-1(tm2255)*	Out-of-frame deletion, GEF domain	Wild type	none
*fax-1(gm27)*^b^	*rgl-1* and 8 other genes deleted	Unc^c^	none
*rgl-1(gk275305)*	nonsense	Wild type	none
*rgl-1(gk275304)*	Missense, R361Q, GEF domain	Wild type	Decreased 2°
*ral-1(tm2760)*	Intron 3 deletion	Sterile^d^	Decreased 2°^d^
*ral-1(tm5205)*	Exons 2 and 3 deletion	Variable, Sterile^e^	Decreased 2°^e^
*ral-1(gk628801)*	Missense, R139H	Wild type	Decreased 2°
*ral-1(re179)*	mNeonGreen::3xFlag::rgl-1	Wild type	none^f^

^a^ Alteration in network signaling determined from phenotype in *let-60(n1046*gf*)* background.

^b^ Documented in [36].

^c^ Deletion of *fax-1* causes an Uncoordinated phenotype [36].

^d^ Intron 2 deletion breaks in the middle of the splice donor site, conferring sterility, reduced 2° induction, and disruption of PAR functions [25].

^e^ Exons 2 and 3 deletion removes GTPase domain sequences, confers sterility, delayed growth and reduced 2° induction [25], this study.

^f^ No effect in *let-60(n1046*gf*)* background.

### Using sensitized genetic backgrounds to evaluate parallel signals in VPC induction

Four signaling cascades–two central and two modulatory–control 1° and 2° fate induction. (Wnt signaling also contributes to 1° induction, but interpretation of Wnt signaling is confounded by addition roles in vulval development: competency of VPCs, polarization of 2° lineages, and fusion of 3° cells to the surrounding hypodermis, failure of which can itself potentiate inductive signals [37–46]. Consequently, Wnt signaling was not included in this analysis.)

To aid understanding of genetic experiments presented in this study, we foreshadow our conclusions by presenting a schematic of the signaling cascades discussed in this study, along with mammalian orthologs of proteins discussed here (Fig 1B and 1C; S1 Fig). Core 1°- and 2°-promoting signals are detectable by direct mutation: since they are necessary and sufficient to induce their respective fates, mutational perturbation of them causes loss or gain of vulval cell types. In contrast, the role of the two modulatory cascades is not revealed through single mutant analysis, but rather requires sensitized genetic backgrounds ([24]; this study). Parallelism has been used extensively by us and others to analyze modulatory signals in VPC fate induction, primarily through use of modifier genetics, *e*.*g*. [5–7, 10, 12, 47, 48].

To analyze such signals, we used *let-60(n1046*gf*)*, a moderately activating G13E mutation analogous to mutations found in a subset of mammalian cancers [49]. In this background, gain and loss of the RAL-1 2°-promoting signal resulted in decrease and increase of ectopic 1° cells, respectively [10, 12]. We have similarly used the *let-23(sa62*gf*)* activating mutation in the LET-23/EGFR [10]. For an under-induced background, we used *lin-45(n2506)* [10]. Through combined use of genetic principles of parallelism and epistasis, we are able to dissect the modulatory signals. We present these principles as a network circuitry diagram (Fig 1C). We have exploited such techniques to delineate a 2°-promoting signaling cascade downstream of RAL-1 [12]. Here we use these techniques to similarly dissect the two roles of RGL-1/RalGEF in 2°- and 1°-promoting activities.

### RGL-1 performs a function in VPC cell fate patterning that opposes its canonical 2°-promoting function

**Fig 2:**
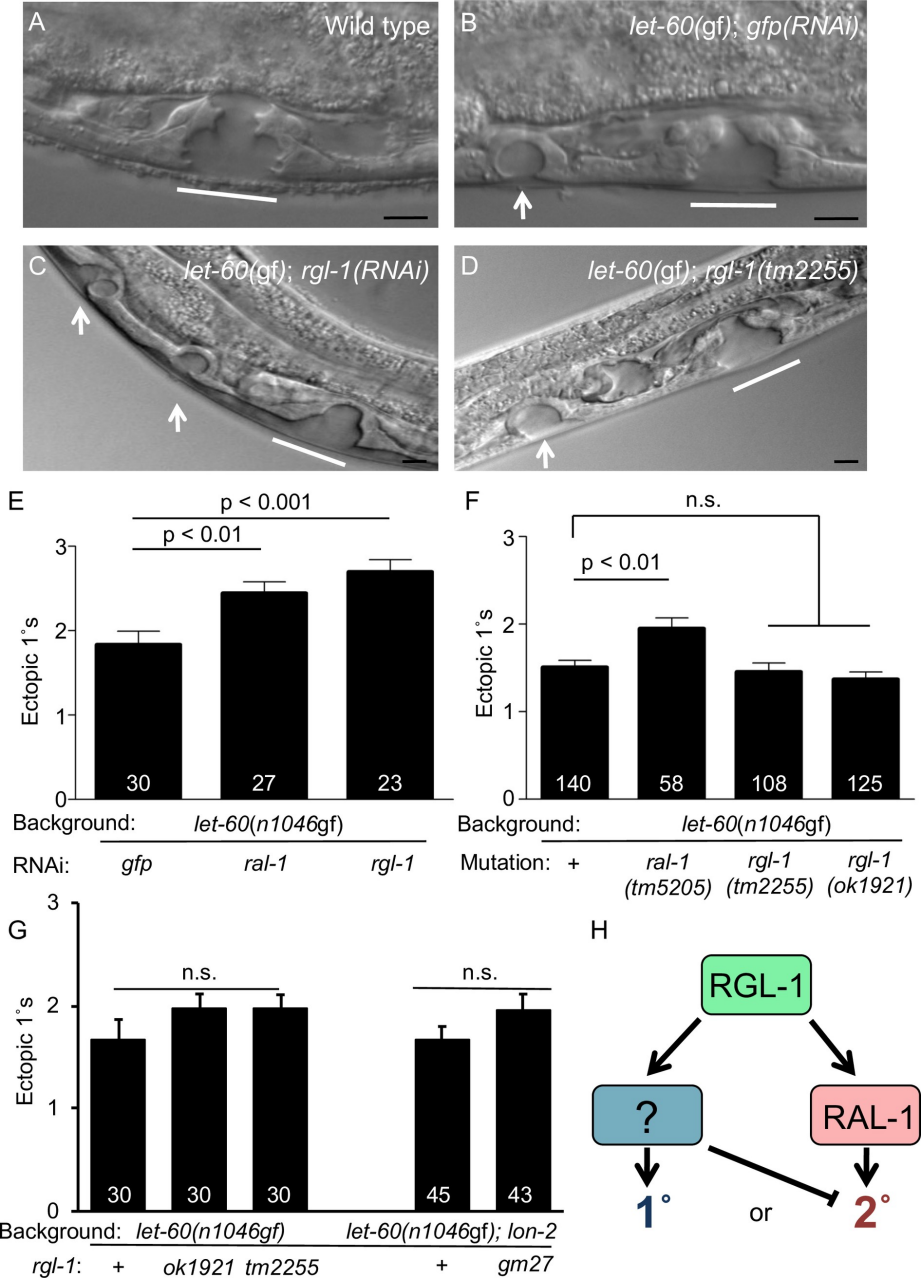
RGL-1 and RAL-1 are functionally non-equivalent in VPC patterning. 1000x photomicrographs of late L4 **A)** wild-type, **B)** *let-60(n1046*gf*)*; *gfp(RNAi)* compared to 600x photomicrographs of **C)** *let-60(n1046*gf*)*; *rgl-1(RNAi)*, and **D)** *let-60(n1046*gf*); rgl-1(tm2255)* animals. White lines = normal 2°-1°-2° L4 vulvae, white arrows = L4 ectopic 1° pseudovulvae. Black scale bars = 5 μm. Anterior is left and ventral down. **E)** RNAi depletion of *rgl-1* and *ral-1* enhance 1° induction relative to *gfp(RNAi)* control. Data are the mean ± standard error of the mean (SEM). For statistical reasons single, non-pooled assays are shown, and white numbers represent animals scored therein. Significance was calculated by Kruskal-Wallis, Dunn test. Data shown were scored concurrently and are representative of 4 independent assays (this study) and 6 prior independent assays [12]. (The *let-60(n1046*gf*)* 1° induction baseline is consistently higher when grown on HT115 bacterially-mediated RNAi food compared to the standard OP50; [10, 12, 48]). **F)** Deletion of *ral-1* but not *rgl-1* enhances ectopic 1° induction by *let-60(n1046*gf*)*. Data shown are representative of 4 assays, each scored concurrently. **G)** Re-constructed strains show the same result: *ok1921*, *tm2255* and *gm27* deletions fail to significantly enhance ectopic 1° induction by *let-60(n1046*gf*)*. Left: Three *n1046*-containing isolates, with and without *rgl-1* mutations and scored concurrently. The concurrently scored MT2124 1° induction baseline was not significantly different from outcrossed lines DV2214 and DV2215 (see Table 1), N = 30 for each, and from assays that showed the most deviation of double mutants from the single mutant, but are still not significantly different. Right: DV2251 *let-60(n1046*gf*)*; *lon-2(e678)* vs. DV2252 *let-60(n1046*gf*)*; *lon-2(e678) gm27* animals scored concurrently but separate from the left group, representative of two assays. **H)** A general model for opposing RGL-1 GEF/RAL-1-dependent and -independent functions (green = bifunctional, blue = 1°-promoting, rose = 2°-promoting; see Fig 1).

We previously showed that depletion of *rgl-1* by RNAi revealed a role of RGL-1 in promoting 2° fate consistent with the established Ras-RalGEF-Ral signal in mammals [10]. We reproduce these RNAi-based experiments here (Fig 2A, 2C and 2E). We also found that *ral-1(tm5205)* confers enhancement of 1° induction in the *let-60(n1046*gf*)* background (Fig 2F), validating our previous results with RAL-1.

To our surprise, analysis of various strong loss or putative null *rgl-1* mutations in the *let-60(n1046*gf*)* background caused no net effect compared to the *let-60(n1046*gf*)* single mutant (Fig 2D, 2F and 2G; see Table 1 for *rgl-1* alleles). Using a 1° fate reporter that indicates adjacent 1° cells in the *let-60(*gf*)* background [6, 10], we found that *rgl-1(RNAi)* but not *rgl-1(tm2255)* significantly increases the occurrence of adjacent 1°s (S2A–S2E Fig). To test that these results are not specific to the *let-60(n1046*gf*)* sensitized background or a 1° over-inducing background, we also assessed the role of deleted *rgl-1* in under-induced background *lin-45(n2506*rf*)* and over-induced background *let-23(sa62*gf*)*. We previously showed that *rgl-1(RNAi)* increased 1° induction in both *lin-45(n2506*rf*)* and *let-23(sa62*gf*)* backgrounds [10]. We found no effect of *rgl-1(tm2255)* in these backgrounds (S2F and S2G Fig).

To summarize, in all of our analyses, *rgl-1(RNAi)* and *ral-1(RNAi)* confer decreased 2°-promoting signaling. In contrast, deletion of *ral-1* but not *rgl-1* decreases induction of 2° fate. Thus, our results are consistent with the working model that RGL-1 performs an additional, Ral-independent function that antagonizes its canonical function, perhaps by promoting 1° fate. The discrepancy between RNAi- and mutational-based analyses is consistent with RGL-1 having different functional thresholds in level of gene product for the two opposing activities, which we have been unable to further investigate.

### Tagged endogenous RGL-1 is expressed ubiquitously, including uniform expression throughout VPC development

**Fig 3:**
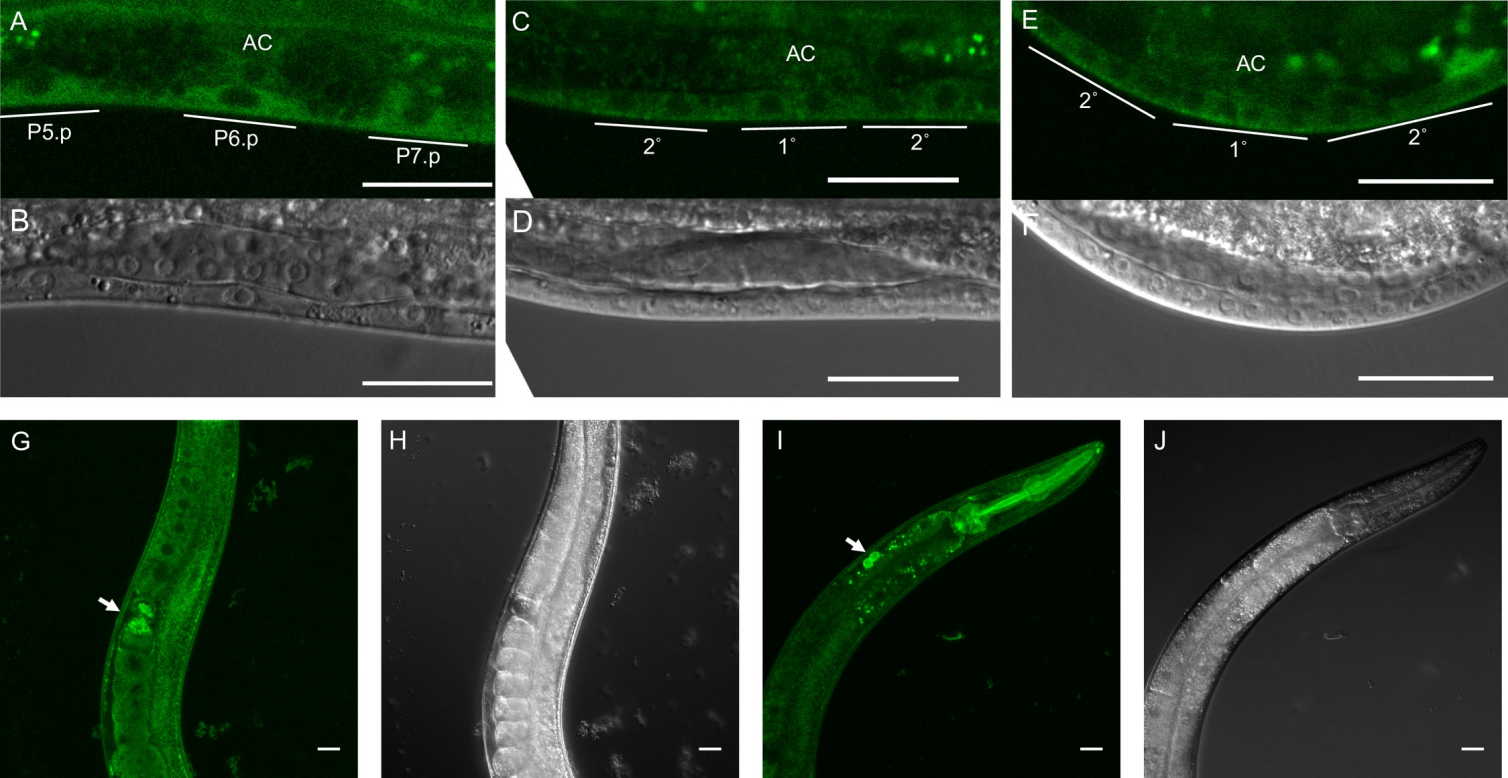
Ubiquitous expression of endogenous tagged RGL-1. **A-F)** We observed CRISPR-tagged endogenous RGL-1 throughout vulval lineages, located primarily in the cytosol (confocal: A, C, E; DIC: B, D, F). **G-J)** We also observed ubiquitous expression of endogenous mNG::RGL-1 in the adult animal. The arrow in **G** indicates sperm in the spermatheca. The arrow in **I** indicates brighter signal from coelomocytes. (confocal: G, I; DIC: H, J.) All scale bars = 20 μm.

We used the self-excising cassette (SEC) approach [50] to tag the endogenous 5’ end of the endogenous *rgl-1* locus with sequences encoding mNeonGreen fluorescent protein (mNG; FP) and a 3xFlag epitope tag (S3A and S3B Fig). We observed uniform signal throughout vulval lineages (Fig 3A–3E). We also observed ubiquitous somatic and germline expression (Fig 3G–3J).

### The *rgl-1* transcriptional fusion is expressed in both 1° and 2° lineages

We previously described a transgenic *ral-1* transcriptional fusion that expressed GFP dynamically over the time course of VPC fate induction [10]. To summarize published results, early in the 3^rd^ larval stage (L3), GFP was expressed consistently in all six VPCs. Later in L3, after induction, GFP expression was excluded from presumptive 1° cells while persisting in presumptive 2° cells. These observations provided a potentially important mechanistic insight: by reducing inferred RAL-1 expression in presumptive 1° cells while retaining expression in presumptive 2° cells, the signaling network attenuates inappropriate RAL-1 activation in presumptive 1° cells, thereby preventing potentially contradictory LET-60 signaling through RGL-1-RAL-1 in presumptive 1° cells.

We similarly tested the expression pattern of a transgene harboring the *rgl-1* promoter transcriptional fusion to GFP, *sEx14985* [51]. As with the *ral-1* promoter transcriptional fusion, the transgenic *rgl-1* reporter was expressed in all VPCs early in L3 (S3C Fig). But, unlike the *ral-1* transcriptional fusion, GFP from the *rgl-1* transcriptional fusion persisted in presumptive 1° cells throughout vulval patterning and proliferation. After the first VPC cell division, GFP expression was consistently higher in 1° relative to 2° cells.

The significance of this change is unclear, since many transcriptional fusions of vulval signaling genes change expression patterns around this time [5–7, 10, 12, 47, 48]; reviewed in [52]. One interpretation is that increased expression of RGL-1 in presumptive 1° cells accounts for resistance to *rgl-1(RNAi)* of this putative 1°-promoting activity of RGL-1.

Our observation with a *rgl-1* transcriptional reporter transgene also contradicts our observation of endogenous tagged RGL-1, where we observed ubiquitous RGL-1 expression (Fig 3 vs S3C Fig). We observed similar discrepancy between dynamic expression from the *ral-1* transcriptional reporter transgene [10] and CRISPR-tagged endogenous RAL-1 [12]. We speculate i) that our promoter fusions lack regulatory sequences that drive ubiquitous expression, but ii) may reflect subtle alterations in expression in VPCs after induction that may shift responsiveness to different signals. We also note that the transcriptional reporter transgene is highly over-expressed, which may alter expression patterns and intensity. Our observations also suggest that CRISPR tags and traditional transcriptional fusion transgene provide complementary views of gene expression.

### RGL-1 performs opposing GEF-dependent and GEF-independent functions in VPC fate patterning

**Fig 4:**
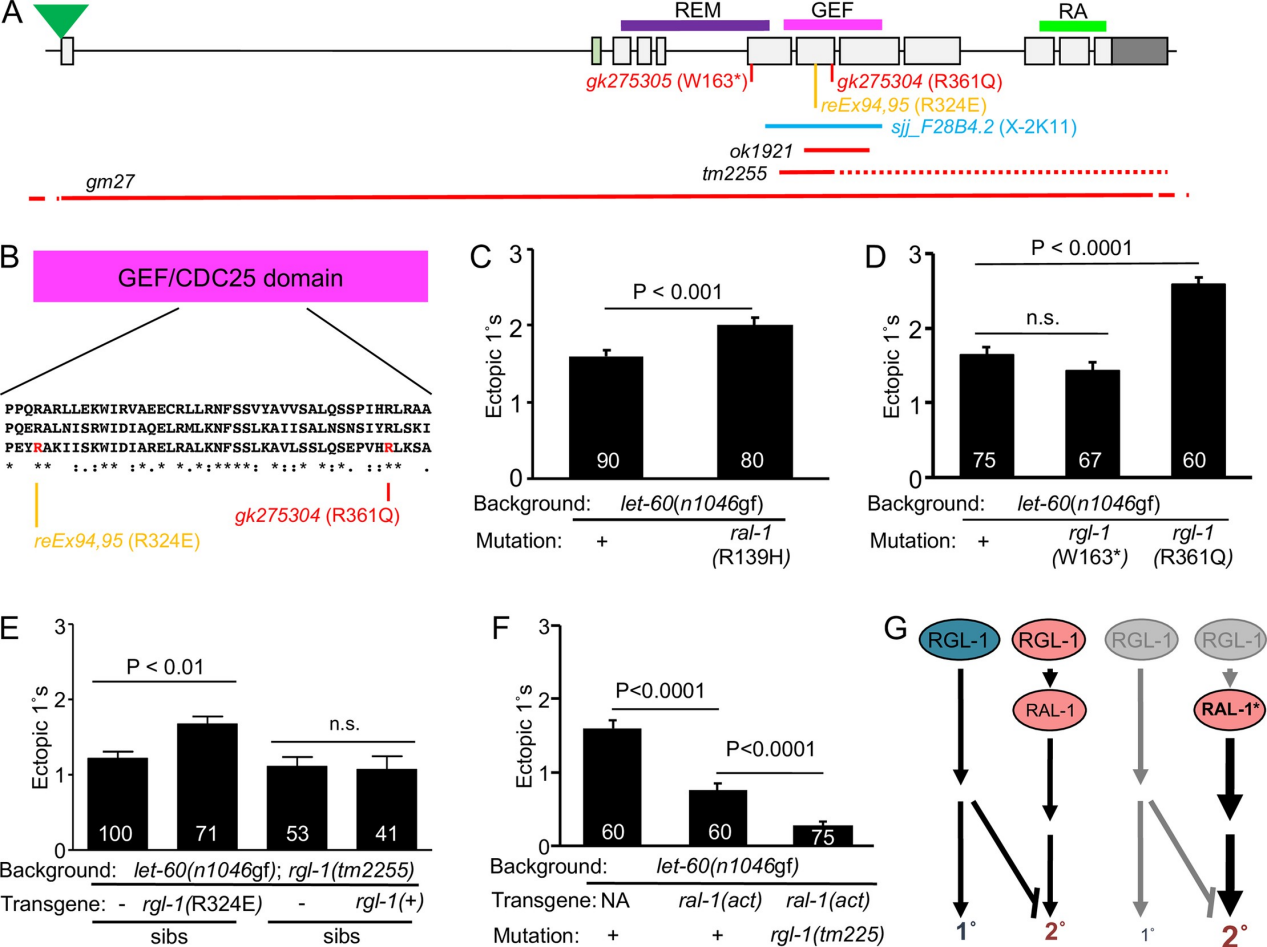
RGL-1 encodes genetically separable functions. **A)** A schematic of the *rgl-1* gene and genetic reagents for this analysis. Green triangle = mNeonGreen::3xFlag tag by CRISPR (S3 Fig). Light green exon 2: by RNAseq this is a rare mRNA species, and the 20 residues coded for by Exon 2 are not conserved among *Drosophila* and mammalian RalGEFs. Purple, pink and bright green lines: REM, GEF and RA (Ras Association) domains (The REM domain is a structural component of some but not all Ras family GEFs). Red *gk* alleles: W163* and R361Q mutations from the million mutation project. Orange: canonical R324E GEF-deficient mutation in transgenes *reEx94* and *reEx95*. Light blue: coverage of bacterially mediated RNAi clone (library location; [54]). Red lines: sequences deleted by *ok1921*, *tm2255* and *gm27* deletions (dotted line indicates that deletion ends out of frame). **B)** An alignment of a portion of the RalGEF domain containing the canonical GEF-deficient R324E and *gk275304* R361Q mutations (top to bottom: Human RGL2, in which the GEF-deficient mutation was validated, fly RalGEF, *C*. *elegans* RGL-1). **C)** The *ral-1(gk628801*rf*)* R139H missense mutation enhances 1° induction in the *let-60(n1046*gf*)* background. Data shown were scored concurrently and are representative of two assays. **D)** *rgl-1* mutation *gk275304* (R361Q) but not *gk275305* (W163*) enhances 1° induction in the *let-60(n1046*gf*)* background. Data shown were scored concurrently and are representative of two assays. **E)** Transgenic rescue of *rgl-1(tm2255)* in the *let-60(n1046*gf*)* background. Transgenic array-bearing animals harboring P_*lin-31*_::*rgl-1* cDNA with P_*myo-2*_::*gfp* co-injection marker and their non-array-bearing siblings were scored. The *reEx94* (shown) and *reEx95* (P<0.006, N = 96 and N = 88) R324E mutant transgenes enhanced 1° induction relative to their non-transgenic siblings, as scored in separate concurrent assays for each array, suggesting that a GEF-independent 1°-promoting activity of RGL-1 functions cell autonomously in the VPCs. (*reEx94 rgl-1(R324E)* mutant transgenic animals failed to respond to *ral-1(RNAi)*, S4C Fig) The *reEx109* (shown) and *reEx110* (also n.s., N = 63 and N = 57) wild-type transgenes failed to alter 1° induction relative to their non-array-bearing siblings, as scored in separate concurrent assays for each array, suggesting that the GEF-dependent 2°-promoting activity of RGL-1 also functions cell autonomously in the VPCs. (*reEx109 rgl-*1*(*+*)* transgenic animals responded to *ral-1(RNAi)*, S4D Fig) **F)** *reIs10[P*_*lin-31*_::*ral-1A(Q75L)*, *P*_*myo-2*_::*gfp]* (“*ral-1(act)*”) suppressed ectopic 1° induction in the *let-60(n1046*gf*)* background; in a single concurrent assay, 1° induction was further suppressed by *rgl-1(tm2255)*, revealing an opposing RGL-1 signal that may be 1°-promoting. Further validation of *reIs10* “*ral-1(act)*” shown in S4B Fig. Data are the mean ± standard error of the mean (SEM). For statistical reasons single, non-pooled assays are shown, and white numbers represent animals scored therein. Significance was calculated by Kruskal-Wallis, Dunn test. **G)** A schematic of the bypass experiment in **F**. Left: canonical and non-canonical RGL-1 activities are opposed and roughly equivalent (in the backgrounds assayed). Right: constitutive, VPC-specific activation of RAL-1 bypasses the GEF activity while also revealing a GEF-independent function of RGL-1, resulting in increased 2°-promoting signal and concomitant loss of 1°-promoting signal, with a net decrease of ectopic 1° cells.

Here we describe a putative reduced function (rf) mutation in *ral-1*. A list of *ral-1* and *rgl-1* mutations is shown (Fig 4A and 4B; Table 1). The Million Mutation Project (MMP) described random sequence identification of a large collection of mutagenized *C*. *elegans* lines, demanding only that mutant animals be viable and fertile over many generations [53]. We analyzed the sole non-synonymous mutation in *ral-1*, *gk628801*, which caused an R139H mutation. Arg-139 is conserved in all Ras family members in metazoans. Outcrossed *ral-1(gk628801*rf*)* single mutant vulvae were superficially wild type (N = 83). In the *let-60(n1046*gf*)* background, *ral-1(gk628801*rf*)* resulted in elevated 1° induction (Fig 4C), consistent with our previously published analysis using *ral-1(RNAi)* and deletion mutations (Fig 2; [10]). Thus, *ral-1(gk628801*rf*)* reduces RAL-1 2°-promoting signaling. Notably, *ral-1(gk628801*rf*)* animals are viable and fertile, in contrast with PAR- and exocyst-associated phenotypes described for deletions *ral-1(tm2760)* and *ral-1(tm5205)* (see above). We speculate that *ral-1(gk628801*rf*)* perturbs GTP-loading required for RAL-1 activation, but does not perturb exocyst- and PAR-dependent RAL-1 functions. This observation is consistent with the observation that RGL-1/RalGEF function is not necessary for exocyst- and PAR-dependent RAL-1 functions, consistent with the idea that GTP-loaded RAL-1 is not necessary for those functions.

Importantly, *ral-1(gk628801*rf*)* confers a vulval induction phenotype similar to a GEF-specific mutation in *rgl-1*. Of 30 total non-synonymous MMP mutations in *rgl-1*, two are likely to perturb function. *rgl-1(gk275304)* causes an R361Q change. Arg-361 is conserved in all CDC25/RasGEF domains. In the *n1046*gf background, *rgl-1(gk275304)* increased ectopic 1° induction (Fig 4D), similarly to *ral-1(gk628801*rf*)* (above). This result is consistent with disrupting GEF domain function and abrogating GTP-loading and activation of RAL-1. *rgl-1(gk275305)* causes a W163* change, which did not alter ectopic 1° induction in the *let-60(n1046*gf*)* background. Both outcrossed single mutant strains were superficially wild type (N = 48 and 61, respectively). We therefore hypothesized that *rgl-1* encodes GEF-dependent and GEF-independent activities.

Using the VPC-specific *lin-31* promoter [55], in the *let-60(n1046*gf*)*; *rgl-1(tm2255)* double mutant background we generated transgenic extrachromosomal arrays expressing VPC-specific RGL-1(+) or RGL-1(R324E), a mutation deficient in GEF catalytic activity in mammalian RalGDS (RalGEF; [56]). Animals bearing the R324E transgenes showed significant increase in ectopic 1° induction compared to non-array-bearing siblings. This result is consistent with rescue of a GEF-independent function of RGL-1. Animals bearing the wild-type transgenes were not different than their non-array-bearing siblings (Fig 4E). Consequently, we propose that RGL-1 performs GEF-dependent and GEF-independent functions that are genetically separable by mutating the GEF domain. We also hypothesize that the GEF-independent RGL-1 activity can function cell autonomously in the VPCs.

Rescue of GEF activity should also rescue the ability of RAL-1 to be activated by the GEF. Hence, transgenic animals expressing VPC-specific RGL-1(+) but not RGL-1(R324E) should restore responsiveness to *ral-1(RNAi*). We evaluated responsiveness of the *let-60(n1046*gf*)*; *rgl-1(tm2255)* background harboring each transgene. *reEx94*/*95* transgenic RGL-1(R324E) animals failed to respond to *ral-1(RNAi)* compared to control *gfp(RNAi)* (S4A Fig; the 1° induction baseline is elevated due to rescue of the putative GEF-independent function shown in Fig 4E). In contrast, *reEx109*/*110* transgenic RGL-1(+) animals restored responsiveness to *ral-1(RNAi*), resulting in increased 1° induction, consistent with VPC-specific rescue of GEF activity and potentially cell autonomy of the GEF-dependent function of RGL-1 (S4B Fig).

### Genetic bypass further reveals a GEF-independent activity of RGL-1

Ectopic 1° induction caused by mutation of *lin-31* was insensitive to perturbation of LET-60-LIN-45-MEK-2-MPK-1 1°-promoting signaling, as expected. But ectopic 1° induction caused by *lin-31(n301)* was enhanced by RNAi targeting *let-60*, *rgl-1*, and *ral-1*. These results indicated that LIN-31/FoxB functions downstream of ERK/MAPK-1 but in parallel to LET-60-RGL-1-RAL-1 [10].

We find that *rgl-1(tm2255)* similarly increased ectopic 1° induction of *lin-31(n301)* animals (S4C Fig). That only the 2°-promoting activity of RGL-1 is revealed by deletion of *rgl-1* in the *lin-31(n301)* background is consistent with the putative non-canonical, GEF-independent signal functioning upstream of LIN-31.

Vulval-specific expression of mutationally active *ral-1* by *reEx24[P*_*lin-31*_::*ral-1(Q75L)*, *P*_*myo-2*_::*gfp)]* suppressed ectopic 1° induction by *let-60(*n1046*gf*) [10]. We used *reEx24* to generate the integrated transgene *reIs10[P*_*lin-31*_::*ral-1A(Q75L*), *P*_*myo-2*_::*gfp]* (see Methods). Similar to *reEx24*, *reIs10[P*_*lin-31*_::*ral-1(Q75L)*, *P*_*myo-2*_::*gfp)]* decreased ectopic 1° induction in the *let-60(n1046*gf*)* background (S4D Fig). Into this background we crossed the *rgl-1(tm2255)* out-of-frame deletion mutation and scored the three strains concurrently. *rgl-1(tm2255)* mutation significantly decreased ectopic 1° induction below the level observed with *reIs10* alone, nearly to wild-type levels (Fig 4F), suggesting that with *reIs10[P*_*lin-31*_::*ral-1(Q75L)*, *P*_*myo-2*_::*gfp)]* we had bypassed the GEF-dependent but not the GEF-independent defect of *rgl-1(tm2255*). We additionally re-derived *n1046-* and *reIs10*; *n1046*-bearing strains and constructed strains with *rgl-1(gk275304)* (R361Q) or *rgl-1(gk275305)* (W163*) instead of the *rgl-1(tm22555)* deletion (S2 Table). Our results were similar to those found in the initial bypass experiment: the R361Q mutation failed to further suppress ectopic 1° induction, while the W163* further suppressed ectopic 1° induction (S4E Fig). We hypothesize that *rgl-1* encodes two antagonistic signals: the GEF- and Ral-dependent 2°-promoting signal, and an unknown antagonistic signal, schematized in Fig 4G.

### RGL-1 interacts with the 1°-promoting PI3K-PDK-Akt cascade

**Fig 5:**
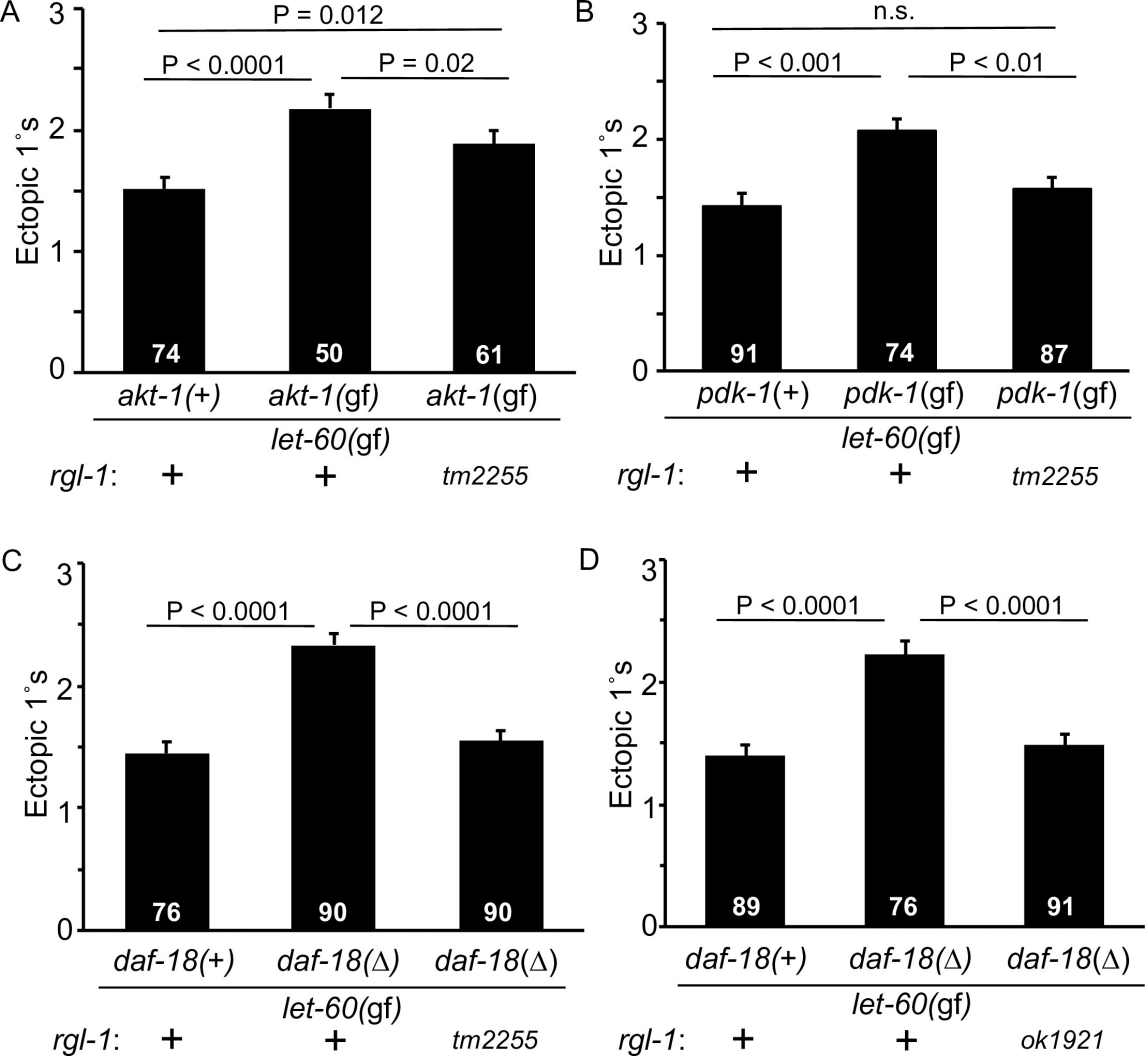
RGL-1 interacts genetically with the 1°-promoting AGE-1/PI3K-PDK-1-AKT-1 cascade. **A)** The constitutively activating *akt-1(mg144*gf*)* mutation increased promotion of 1° fate in the *let-60(n1046*gf*)* background, and this effect was partially blocked by *rgl-1(tm2255*), though the triple mutant was not suppressed to the double mutant baseline level. Animals were scored concurrently. These results were replicated with a re-built strain (S5A Fig) and the strain with *rgl-1(ok1921)* (S5B Fig). Data are the mean ± standard error of the mean (SEM). For statistical reasons single, non-pooled assays are shown, and white numbers represent animals scored therein. Significance was calculated by Kruskal-Wallis, Dunn test. **B)** The constitutively activating *pdk-1(mg142*gf*)* mutation increased promotion of 1° fate in the *let-60(n1046*gf*)* background, and was completely suppressed to baseline level by *rgl-1(tm2255)*. Animals were scored concurrently and are representative of two assays. **C, D)** Mutation of the negative regulatory PTEN ortholog by *daf-18(ok480)* similarly increased 1° induction, and was completely suppressed to baseline level by *rgl-1(tm2255)* **(C)** and *rgl-1(ok1921)* **(D)**. Animals for each were scored concurrently and scoring was repeated once, with the same general results. *rgl-1(gk275305)* (nonsense; S5C Fig) but not *rgl-1(gk265304)* (GEF; S5D Fig) suppressed this *ok480* enhancement, suggesting that the pertinent RGL-1 activity is GEF-independent/non-canonical.

Mammalian RalGDS binds to PDK and Akt1 in cultured cells, possibly functioning as a scaffold for PDK and Akt [26, 27]. The AGE-1/PI3K-PDK-1 signal has been described as promoting 1° fate in VPC fate patterning [24], but AKT-1 was not implicated downstream of this process, potentially because of redundancy of AKT-1 and AKT-2 in *C*. *elegans* [57, 58]. Previously, a gain-of-function mutation in AKT-1 was tested in a genetic background with reduced 1° induction, not *let-60(n1046*gf*)*. No effect was found, leading to the model that AKT-1 did not contribute to vulval induction [24]. We re-evaluated the *akt-1(mg144*gf*)* mutation in the *let-60(n1046*gf*)* background and observed significant increase in ectopic 1° induction (Fig 5A). To corroborate previously published results, we assessed the impact of both activated AKT-1 and PDK-1 in the hypo-induced *lin-45(n2506*rf*)* background: *akt-1(mg144*gf*)* did not alter the hypo-induced *lin-45(n2506*rf*)* phenotype, but *pdk-1(mg142*gf*)* did suppress the 1°-induction defect (S5C and S5D Fig). This result, coupled with earlier analysis [24], suggests that the canonical AGE-1/PI3K-PDK-1/PDK-AKT-1/Akt cascade functions to promote 1° vulval fate. Perhaps activated AKT-1 is not sufficient to promote 1° fate in an under-induced background because it functions redundantly with AKT-2, while the activated upstream PDK-1 is sufficient (see below).

Including the *rgl-1(tm2255)* mutation in the *let-60(n1046*gf*)*; *akt-1(mg144*gf*)* background significantly suppressed ectopic 1° induction, but not to the baseline of the *n1046*gf single mutant (Fig 5A). Since we observed intermediate suppression, we constructed this strain twice and observed a similar result (S5A Fig). We also reproduced this result with *rgl-1(ok1921)* and observed similar intermediate strength suppression that remained significantly above the baseline of the *let-60(n1046*gf*)* single mutant (S5B Fig).

We further tested the relationship of RGL-1 with the rest of the PI3K cascade. Mutational activation of PDK-1 via the *pdk-1(mg142*gf*)* mutation also increased ectopic 1° induction in the *let-60(n1046*gf*)* background. This effect was completely suppressed by *rgl-1(tm2255)* (Fig 5B), suggesting a quantitatively detectable difference between the epistatic relationships of PDK-1 and AKT-1 with RGL-1. Genetic disruption of DAF-18/PTEN, a negative regulator of this cascade in *C*. *elegans* in general [59, 60] and VPC fate patterning in particular [24], increases ectopic 1° induction in the *n1046*gf background. We found that *rgl-1(tm2255)* completely blocked this effect (Fig 5B). The nonsense *rgl-1(gk275305)* but not the putative GEF-deficient *rgl-1(gk275305)* mutation similarly suppressed (S5E and S5F Fig), further supporting the hypothesis that the putative RGL-1 1°-promoting is GEF-independent.

While mammalian RalGDS bound directly to PDK, RalGDS bound indirectly to Akt through the intermediary scaffold, JIP (JNK Interacting Protein; [26, 27]). Deletion the sole *C*. *elegans* JIP ortholog, JIP-1 (S5I Fig) or RNAi depletion of JIP-1 (S5G Fig) suppressed the ectopic 1° phenotype of the *daf-18(ok480) let-60(n1046*gf*)* double mutant, consistent with JIP-1 collaborating with RGL-1 to scaffold PDK-1 and AKT-1 1°-promoting signaling. RNAi depletion of both *pdk-1* and *rgl-1* similarly suppressed the enhanced 1° induction of *daf-18(ok480) let-60(n1046*gf*)* (S5H Fig). However, the *jip-1* deletion allele enhanced *n1046*gf alone while suppressing *daf-18(ok480) n1046*gf. (S5I Fig). We speculate that JIP-1 functions in the PI3K cascade, but may also function elsewhere in VPC fate patterning, perhaps in its canonical role as a scaffold for JKK and JNK MAP kinases. Further investigation of the role of JIP-1 is beyond the scope of this study.

A common target of the *C*. *elegans* PI3K-Akt cascade is inhibition of the DAF-16/FoxO transcription factor [61, 62]. We tested the role of *daf-16* alleles *mu26*, *mu86* and *mgDf47* in different backgrounds, with inconclusive results. Consequently, we were unable to determine the role, if any, of DAF-16/FoxO in VPC fate patterning. Perhaps AKT-1 has a target other than DAF-16 in this system, or the diversity of functionally distinct DAF-16 isoforms results in inconclusive results with *daf-16* mutations that perturb all isoforms [63, 64].

Taken together, these genetic epistasis experiments, using an assay of parallelism with *let-60(n1046*gf*)*, are consistent with RGL-1 contributes to the AGE-1/PI3K-PDK-1/PDK-AKT-1/Akt cascade in VPC fate patterning. Alone among the genetic tools used, the gain-of-function mutation in the downstream AKT-1 was only partially suppressed by *rgl-1(tm2255*), while the effect of other Akt cascade activators was completely suppressed by *rgl-1(tm2255*). Perhaps RGL-1 is essential for the PDK-1 1°-promoting signal but only partially required for the AKT-1 1°-promoting signal. We note that mammalian Akt is activated via parallel mechanisms: phosphorylation by upstream PDK and binding of PIP_3_, resulting in recruitment to the plasma membrane and activation [65]. If RGL-1 functions as a scaffold for PDK-1 and AKT-1, its deletion would be expected to result in reduction of the PDK-1 phosphorylation of AKT-1 but not PIP_3_-dependent recruitment of AKT-1 to the plasma membrane. Thus, the pattern of suppression by mutated RGL-1 is consistent with RGL-1 functioning as a scaffold for PDK-1-AKT-1 signaling in 1° fate induction. However, we cannot rule out the possibility of parallelism between a GEF-independent RGL-1 activity and the AKT-1 cascade, or RGL-1 functioning in a bifurcated cascade downstream of AKT-1 that is not revealed by the mechanism of constitutively activated PDK-1 or DAF-18/PTEN deletion upstream.

### Deletion of RGL-1 decreases fidelity of VPC patterning

Based on a relatively small sample size, RGL-1 deletions cause no gross VPC patterning defects, consistent with both AKT-1 and RAL-1 cascades being modulatory, not central. We hypothesized that RGL-1 orchestrates activation of these two potentially opposing cascades–Akt output to presumptive 1° cells and Ral output to presumptive 2° cells–to improve robustness of the VPC developmental system in response to environmental stressors. A previous study investigated the impact on VPC fate patterning of environmental stressors: the N2 baseline error rate under laboratory conditions was 0.2% [66]. We similarly investigated the impact of environmental stressors of starvation, heat, and osmotic stress, compared to non-stressful conditions, on wild-type, *rgl-1(ok1921*), and *rgl-1(tm2255)* animals.

**Fig 6:**
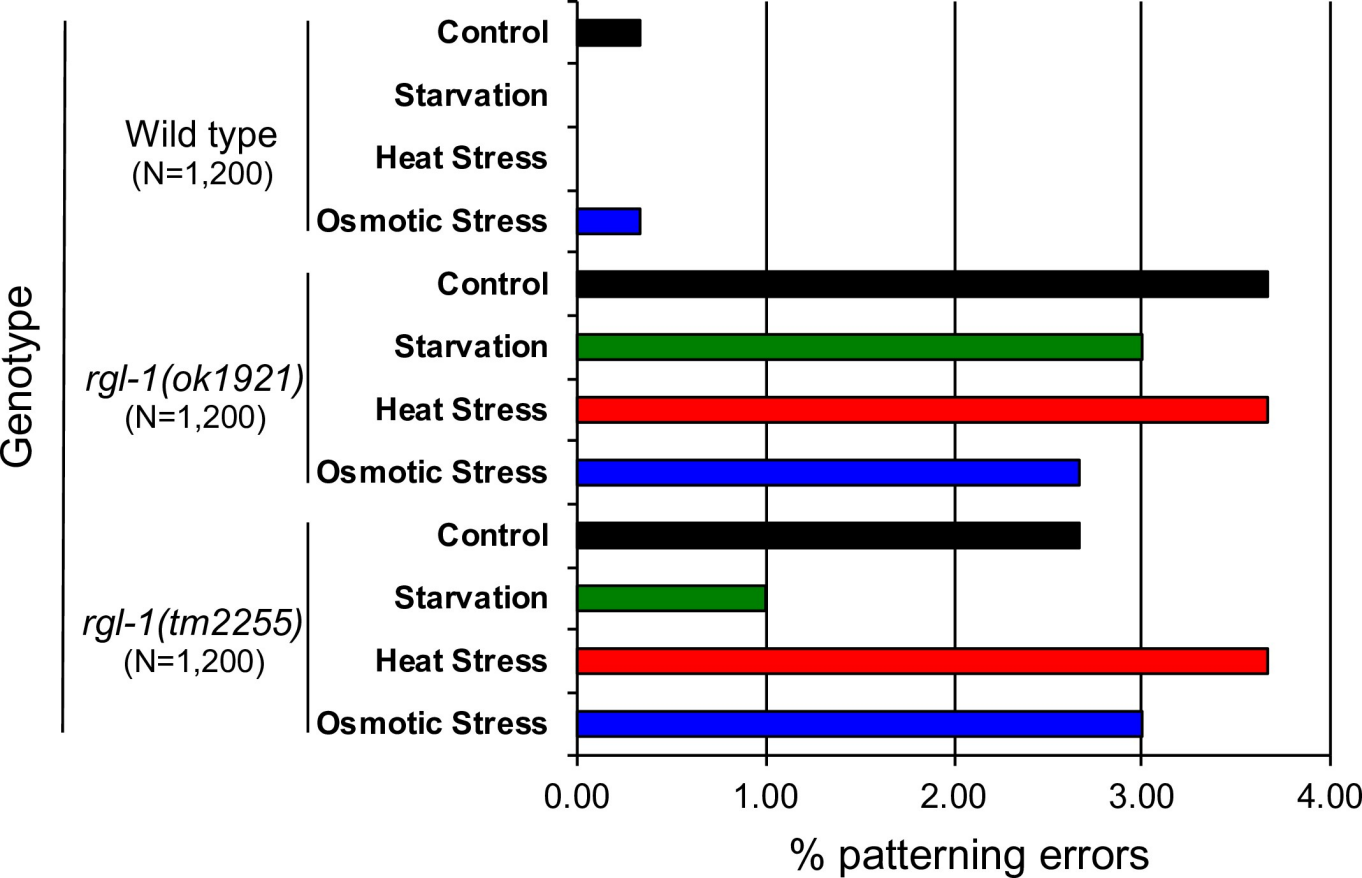
Deletion of RGL-1 increases patterning errors but not susceptibility to environmental stress. *rgl-1* deletions *ok1921* and *tm2255*, introgressed to the wild type for eight generations (DV2696, DV2697), caused 15-fold increased patterning defects compared to the wild type. (N = 1,200 per genotype, pooled from 300 for each condition.) P<0.0001 for each genotype, P>0.05 between environmental conditions.

We evaluated 300 animals per genotype under each condition, totaling 1,200 animals per genotype and 3,600 animals overall (Fig 6A). Similar to previous results [66], wild-type animals exhibited very low error rates (0.0–0.3%), not only in control but also in stressful conditions. In contrast, the error rate in VPC patterning of both *rgl-1* mutants was substantially increased across all experimental environments (1.0–3.7%), including control environments, in which patterning error rates were increased more than 15-fold relative to the wild type. These results indicate an environmentally-insensitive increase in error rate in the absence of functional RGL-1. Consequently, we propose that RGL-1 does not impact robustness in response to environmental stressors. Rather, we hypothesize that RGL-1 mitigates stochasticity within the complex signaling network that regulates VPC fate patterning. This observation represents intriguing and unprecedented insight into the role of signaling networks in developmental fidelity.

## Discussion

We previously showed that LET-60/Ras-RGL-1/RalGEF-RAL-1/Ral signaling promotes 2° VPC fate in support of the necessary and sufficient LIN-12/Notch. Here we analyze two facets of RGL-1 function in *C*. *elegans*. First, in marked contrast to RAL-1, we find that deletion of RGL-1 does not overtly perturb development or fertility. This observation contradicts the expectation from mammalian cell culture: since *C*. *elegans* RAL-1 is essential and required for exocyst-associated functions in multiple organisms, the expectation was that RGL-1 would be similarly required. We hypothesize either that GTP-loading of RAL-1 is not required as part of its function in the exocyst complex, or that a thus far unknown RalGEF functions redundantly with RGL-1 in exocyst-related by not 2°-promoting signals.

Second, we find evidence that RGL-1 performs opposing GEF-dependent and GEF-independent functions that promote fidelity of VPC patterning. Our genetic epistasis results are consistent with RGL-1 functioning in the PDK-1-AKT-1 1°-promoting signal. Our results raise the interesting possibility of opposing and inessential modulatory cascades functioning to combat stochasticity in a signaling network.

### RAL-1 but not RGL-1 is essential for development

RAL-1 was previously implicated in essential developmental events in *C*. *elegans* [10, 25]. Yet despite the ostensible linearity of the Ras-RalGEF-Ral signaling model in mammals [14, 23], we unexpectedly found that RGL-1 is not essential. Given that deletion of neither the GEF nor the GAP disrupts exocyst-dependent developmental events but deletion of RAL-1 does so, we hypothesize that the RAL-1 role as a membrane tether for the exocyst is independent of nucleotide-bound state. This hypothesis does not imply that there is no activation-dependent alteration of exocyst function by RAL-1, merely that such is not essential for the described developmental events.

An alternative hypothesis is that other GEFs function redundantly with RGL-1 to regulate the GDP/GTP cycle of RAL-1. We find that RGL-1 and RAL-1 are required for the 2°-promoting signal. But this other GEF would need to be specific for RAL-1 association with the exocyst and not signaling to promote 2° fate. While we cannot exclude this possibility, no GEFs fitting that role have been described. RalGPS, a mammalian Ral-selective GEF that contains SH3 and PH domains, is not encoded in the *C*. *elegans* genome; [10], nor is TD-60/RCC2, similar to the Ran GEF RCC1 that controls nuclear import/export, which has been proposed to be an atypical GEF for RalA [67].

### RGL-1 performs genetically separable and opposing functions in VPC fate patterning

To our surprise, we found that deletion alleles of *rgl-1* caused no net alteration of the balance of 1° and 2° VPCs in the *let-60(*gf*)* background. By genetic analysis, RGL-1 performs its canonical role in promoting 2° fate as a signaling intermediary between LET-60/Ras and RAL-1 ([10]; this study), a role that mirrors extensive biochemical and cell biological evidence from mammalian cell culture (reviewed in [14, 23]). Here, further genetic analysis reveals an additional, unexpected role for RGL-1, that of a non-canonical signaling participant opposing the canonical role as an activator of RAL-1. This non-canonical role apparently counteracts or “cancels out” the canonical role. In sensitized backgrounds, putative null mutations have no net effect on the sensitive balance between 1° and 2° VPC fates. We do not intend to imply that opposing functions are “equal”, only that in our assays they appear to counter-balance each other with approximate equivalency. The observation that deletion of RGL-1 increased the basal error rate of patterning 15-fold supports the idea that RGL-1 serves an important function, that of fidelity.

In mammalian cell culture RalGEF-Ral and PDK-Akt cascades worked in concert [26]. Yet mammalian RalGEF-Ral signaling has also been found to oppose canonical Ras effectors in cell culture [68]. We speculate that the relationship between these cascades may depend on cell context, and in the case of vertebrates, which paralog of RalGEF is expressed.

RGL-1 is not the first protein in the VPC fate patterning network found to be bifunctional and promote both 1° and 2° fates. Depending on signaling dose and via mechanisms we do not understand, LIN-3/EGF-LET-23/EGFR signaling was found to promote both 1° and 2° fates, resulting in the graded signal model [8, 9, 69]. We found that LET-60/Ras can promote 1° or 2° fate depending on its use of effector, LIN-45/Raf or RGL-1/RalGEF [10, 11]. Yet the situations are not equivalent. LIN-3, LET-23, and LET-60 are essential for vulval induction: strong loss results in complete absence of 1° fate induction and hence a vulvaless phenotype [70–73], and thus their roles in 2° fate induction were teased out only in sensitized backgrounds or special assays [8–10]. In contrast, RGL-1 functions are solely modulatory and dispensable for 1° and 2° fate induction, permitting dissection of its balanced and opposing functions. Furthermore, deletion of RGL-1 does not alter any other known developmental events.

### Linking opposed signaling cascades and mitigating development noise

To occur with high fidelity, the VPC fate patterning system must strictly define developmental fields in response to an initial point source of LIN-3/EGF ligand. In other words, it must generate the precise 3°-3°-2°-1°-2°-3° pattern with 99.8% accuracy [66] without mis-specified or ambiguous fates that might block mating and egg laying, which would negatively impact reproductive fitness. A critical question, then, is how the programming of signal transduction networks decreases the potential for errors (developmental stochasticity or “noise”). Previously, the fidelity of VPC fate patterning was thought to be a property that emerges from the combination of three mechanisms: 1) sequential induction sets up the basic pattern, 2) graded signal collaborates with Sequential Induction to more precisely sculpt the initial pattern, and 3) mutual antagonism serves to exclude potentially conflicting signals from cells that are initially specified, thus preventing assumption of ambiguous fates (reviewed in [52]). We speculate that the roles we describe here for RGL-1 define a fourth method that is woven into the other three: orchestration of two modulatory cascades to more sharply demarcate fates as a function of the VPC’s spatial relationship to the AC and other VPCs.

## Methods

### *C*. *elegans* handling and genetics

Nomenclature is as described [74, 75]. All strains were derived from the N2 wild type. Except where noted, animals were cultured on NGM agar plates with OP50 bacteria on at 20°C [76]. Strains used are shown in S1 Table. Data were analyzed with GraphPad Prism software (GraphPad Software Inc., La Jolla, CA).

PCR primers are listed in S2 Table. For single animal genotyping PCR (Taq PCR Master Mix, Qiagen) reactions were run concurrently with +/+, m/+ and m/m controls. Each PCR genotype was double-checked after completion of the strain construction. For newly analyzed mutations, PCR products were sequenced to confirm break points. *rgl-1(ok1921)* was detected by triplex PCR using primers DJR614/615/616 (Tm: 59°C, 35 cycles), resulting in 366 bp (wild type) and 233 bp (*ok1921*) bands (S6 Fig). Point mutations in *rgl-1* were tracked in *trans* to *ok1921*. Early constructions detected *rgl-1(tm2255)* by triplex PCR using primers TZ20/DJR614/DJR615 (Tm: 58°C, 35 cycles), resulting in 913 bp (wild-type) and 595 bp (*tm2255*) bands (S7 Fig). Later constructions detected *rgl-1(tm2255)* by triplex PCR using primers FSM7/8/9 (Tm: 58°C, 35 cycles), resulting in 509 bp (wild-type) and 254 bp (*tm2255*) bands. *pdk-1(mg142*gf*)* was amplified by primers DRC1/2 (Tm: 60°C, 35 cycles) to generate a 426 bp band, then digested with Hpa II (NEB; 5 units added to total reaction volume doubled with water with NEB buffer #1 to 0.5x total, digested overnight at 37°C). The band from the wild-type allele was digested to yield 126 and 300 bp bands, while the *mg142* lesion abolishes the Hpa II site. *daf-18(ok480)* was detected by triplex PCR using primers FSM4/5/6 (Tm: 54°C, 35 cycles), resulting in 388 bp (wild-type) and 216 bp (*ok480*) bands. For this study, *dpy-9(e14)* was used as a balancer for *daf-18(ok480)*, and the *ok480* genotype confirmed after construction. *akt-1(mg144*gf*)* was balanced during strain constructions by *dpy-11(e224) unc-76 (e905)*. *ral-1(gk628801*rf*)* was detected by amplification with primers DJR778/779 (Tm = 59.9°C, 35 cycles) to generate a 250 bp band and digested with HpyCH4 IV (NEB; 5 units added to total reaction volume doubled with water with NEB buffer #1 to 0.5x total, digested overnight at 37°C). The band from the wild-type allele was digested to yield 122 and 128 bp bands, while the *gk628801* lesion abolishes the HypCH4 IV site.

Transgenic extrachromosomal array *reEx24[P*_*lin-31*_::*ral-1(Q75L)*, *P*_*myo-2*_::*gfp)]* [10] was integrated by irradiation of late L4 animals using a Stratalinker (Stratagene) at dose of 12 mJ/cm^2^. 451 F2 progeny were screened for integration to obtain *reIs10[P*_*lin-31*_::*ral-1(Q75L)*, *P*_*myo-2*_::*gfp)]*. *reIs10* was mapped to the region of Chromosome I +5.

### VPC induction assays

VPC induction was analyzed by DIC/Nomarski optics (Nikon eclipse Ni with images captured using NIS-Elements AR 4.20.00 software) in late L4 animals on an agar pad (molten 3% NG agar with 5 mM sodium azide) in a 5 μl drop of M9 buffer. Ectopic pseudovulvae were scored as invaginations at 600x or 1000x. Ectopic 1° vulval induction index, from 0 to 3 ectopic 1°s, is described elsewhere [10, 12]. Under-induced backgrounds were scored as total VPCs induced, typically 0–3 (in under-induced backgrounds the entire vulva is frequently uninduced). To summarize, we scored the wild-type vulval induction based on the stereotypical 2°-1°-2° lineages centered on the AC at the A-P midpoint of the gonadal primordium (forming the “Christmas Tree” or “Stanley Cup” shape). In the *let-60(n1046*gf*)* and *let-23(sa62*gf*)* backgrounds, the morphology of ectopic 1° cells generally conformed with the described “cap” structure where the entire 1° lineage has pulled away from the cuticle [9]. We did not observe ectopic pseudovulvae with the characteristic asymmetrical “beret” lineage of 2° lineages, where one side remains attached to the cuticle.

As we have described previously [10, 12, 48], we occasionally observed drift of the severity of the *let-60(n1046*gf*)* but not *let-23(sa62*gf*)* Muv phenotype. Consequently, we employed a stringent protocol for all VPC induction scoring experiments. We analyzed parental MT2124 and outcrossed strains harboring the *n1046*gf single mutant, and established that the baseline of undrifted strains averaged 1.2–1.5 ectopic 1°s. (When grown on bacterially mediated RNAi food source HT115, induction was consistently higher: 1.5–1.8; [10, 12, 48]). We additionally always worked with freshly thawed or chunked strains; our animals were always freshly derived from a cross or thaw. For all strains harboring the *n1046*gf mutation we always employed stringent scoring criteria: the *n1046*gf single mutant was scored first, and experiments deviating from the aforementioned expected range of induction were discarded. Additionally, we only compared genotypes that were scored concurrently, thus minimizing variation from assay to assay.

### DIC, epifluorescence and confocal microscopy

For epifluorescent imaging, animals were mounted in 2 mg/ml tetramisole/M9 buffer and visualized using a Nikon Eclipse TE2000U microscope equipped with a DVC-1412 CCD camera (Digital Video Camera Company), with Hamamatsu SimplePCI acquisition software. Confocal images were captured by A1si Confocal Laser Microscope (Nikon) using NIS Elements Advanced Research, Version 4.40 software (Nikon).

### Bacterially mediated RNA interference

RNAi was performed as described previously [10, 12] with HT115 bacterial host [77]. RNAi plasmids used were: pREW2 (*luciferase/luc*; [12]), X-2K11 (*rgl-1*), III-7M13 (*ral-1*), I-1K04 (*pop-1*), and *gfp* [10]. Each RNAi clone was sequence verified. Bacteria were grown on NGM plates supplemented with 50 μg/ml carbenicillin and 1 mM IPTG. Bacteria were grown (but not overgrown) overnight, without antibiotic selection. 80 μl of fresh culture was seeded on plates on day 1, grown overnight, L4 animals were added on day 2, transferred to a fresh plate on day 3, and scored on day 5.

### Plasmid subcloning and transgene generation

Using primers RGL-1F and RGL-1R, the *rgl-1a* cDNA was amplified from clone yk643d11, and digested with Bgl II and Not I. This isoform lacks exon 2, which by RNAseq data is rare (Wormbase WS263), yet still rescues, arguing that exon-2 is not required for vulval signaling. Plasmid vector pB255, which contains the *lin-31* promoter and additional regulatory sequences and drives expression in VPCs [55], was digested with Bgl II and Not I to receive the *rgl-1* insert. The putative R324E GEF-deficient mutation was introduced by PCR with Pfu Turbo using primers KM1 and KM2. The resulting plasmids, P_*lin-31*_::*rgl-1(*+*)* and P_*lin-31*_::*rgl-1(*R324E*)*, were injected at 5 ng/μl along with co-injection marker pPD118.33(P_*myo-2*_::*gfp*) at 5 ng/μl into strain DV2190 *let-60(n1046*gf*)*; *rgl-1(tm2255)* to generate arrays *reEx109* and *reEx110* (*rgl-1(+)*) and *reEx94* and *reEx95* (*rgl-1(R324E)*), which express wild-type and GEF-deficient RGL-1, respectively, specifically in VPCs.

### CRISPR/Cas9-dependent genome editing

*rgl-1(re179[mNeonGreen*::*3xFlag*::*rgl-1])* was generated using the positive-negative selection self-excising cassette method [50]. The repair template for *rgl-1* 5’ tagging was generated by Gibson Assembly (NEB) with digested target SEC vector pDD268, and ~500 bp of homology arms amplified from genomic DNA by Q5 polymerase (NEB). We used two sgRNA sequences: (#1) 5’-ACACCTTCGTATCCTTGTGGCGG-3’ and (#2) 5’-GGTCTGAGTTCTTCTGACGATGG-3’ (PAMs underlined), and hence generated two targeting vectors and one repair template. Repair template (20 ng/μl), sgRNA-Cas9 #1 (25 ng/μl), sgRNA-Cas9 #2 (25 ng/μl) and injection marker P_*myo-2*_::*mCherry* (2.5 ng/μl) were microinjected into wild-type animals. Genotyping and sequencing of *rgl-1* 5’ CRISPR tagging was performed with HS125/126/127 (Tm = 54°C). Validation was performed by western blotting using monoclonal anti-Flag antibody (Sigma-Aldrich F1804; 1:2000), monoclonal anti-α-tubulin antibody (Sigma-Aldrich T6199; 1:2000) and goat anti-mouse secondary antibody (MilliporeSigma 12–349; 1:5000).

For reasons unknown, all seven *rgl-1* CRISPR alleles generated harbored mutations. Repair templates were re-checked by sequencing to confirm that sequences were wild type. DV3225 *rgl-1(re179[mNeonGreen*::*3xFlag*::*rgl-1])* harbored only promoter mutations (C insertion at -614, C deleted at -375, C to T at -395) and so was selected for further analysis. Analysis of the ModEncode database showed no peaks of promoter occupancy at these sites. *rgl-1(re179[mNeonGreen*::*3xFlag*::*rgl-1])* had no effect on 1° induction in the *let-60(n1046*gf*)* background (P = 0.57 between strains with and without the *re179* insertion, N = 86 and 78, respectively).

### Assessment of patterning error rate with environmental insults

The vulval cell lineages and Pn.p fates (and errors) were assessed in early to mid L4 individuals as previously described [66, 78]. Nematode populations of N2 wild type and the two *rgl-1* mutations were kept in identical environmental conditions for at least three generations prior to experiments. Animals were age-synchronized by hypochlorite treatment and liquid arrest for ~24 hours, then randomly allocated to the four experimental environments: control (20°C), heat stress (29°C), osmotic stress (250 mM NaCl NGM plates, 20°C; [79]), and starvation (20°C; [80]). In starvation conditions, L1 larvae were cultivated on standard NGM plates until they reached the mid L2 stage, at which point they were transferred to unseeded NGM plates containing 1 mg/ml of ampicillin to prevent bacterial growth. After 48 hours, starved animals were transferred to regular NGM plates seeded with *E*. *coli* OP50 and the vulval phenotype was scored when animals had reached the early or mid L4 stage [80].

## Supporting information

S1 Fig Signaling network comparisons between humans and *C*. *elegans*. Signaling relationships in **A)** Mammalian carcinomas and **B)** *C*. *elegans* VPC fate patterning. Historically, mammalian interactions have been shown directly, while *C*. *elegans* interactions were deduced from a combination of phenotypes, genetic epistasis, and inferences from biochemical relationships among mammalian orthologs. Color coding is the same as in other figures: blue = 1°-promoting, rose = 2°-promoter, dark = necessary and sufficient signal, light = modulatory signal. Green = both 1°- and 2°-promoting (rather than green, RGL-1 is shown in two places, with a green two-headed arrow denoting possible dual function in both non-canonical 1°-promoting and canonical 2°-promoting roles). Activation of *C*. *elegans* AGE-1/PI3K by a receptor other than DAF-2, or by LET-60/Ras, has not been suggested in the literature, and hence is indicated by dotted lines. The interactions between RGL-1, PDK-1 and AKT-1 are inferred from genetic relationships in this study, and have not been shown directly. JIP-1, a potential intermediary between Akt and RalGDS/RalGEFs inferred from mammalian biochemical analyses in the Feig lab, is not shown.
(TIF)

S2 Fig RGL-1 controls cell fate decision. **A)** Percent Pn.px-staged *let-60(*gf*)* L3 larvae with CFP-positive lineages neighboring the P6.p lineage (P5.p or P7.p derived) with *luc(RNAi)* vs. *rgl-1(RNAi)*. Shown are average percentages of animals with adjacent 1° cell fate. **B)** Percent Pn.px-staged *let-60(*gf*)* L3 larvae with CFP-positive lineages neighboring the P6.p lineage (P5.p or P7.p derived) with *rgl-1(+)* or *rgl-1*(*tm2255*Δ). Y axis is percent adjacent 1°s, white numbers in bars are number of animals scored per genotype. **C-E)** Expression of the 1° fate reporter *arIs92* P_*egl-17*_*∷cfp-lac*Z in VPC daughters. Overlaid DIC and CFP fluorescence images of **C)** *let-60*(*n1046*gf); *luc(RNAi)*, **D)** *let-60*(*n1046*gf); *rgl-1(RNAi)* and **E)** *let-60(n1046*gf); *rgl-1*(*tm2255*Δ) at the Pn.px stage. The black bar indicates P6.px and white bar indicates P7.px cells. **F)** Hypo-induced *lin-45(n2506)* background with and without *tm2255*. Y axis is total induced VPCs (0 = vulvaless, 3 = normal wild-type vulva.) White numbers are number of animals assayed. **G)** *let-23(sa62*gf*)* with and without *tm2255*. Y axis is mean ectopic 1° induction. Error bars show S.E.M. P value calculated via Mann-Whitney test.
(TIF)

S3 Fig VPC expression pattern of the *rgl-1* transcriptional GFP fusion over time. **A)** SEC positive-negative selection strategy for tagging *rgl-1* with CRISPR. **B)** Western blot validation of the *rgl-1* tag before (left) and after (right) SEC excision. The expected band of 129 kD (RGL-1+mNG+3xFlag) was observed. 55 kD α-tubulin loading control is shown below. **C)** We used a combination of DIC analysis of VPCs and migration of the gonadal distal cells for staging to characterize the dynamic pattern of GFP expression from *sEx14985* [51] over time. Initial expression in naïve VPCs is uniform. Around the time of induction, expression is restricted to presumptive vulval lineages. Later expression, after the first cell division, remains higher in 1° than 2° linages. Later stages show low levels of expression in surrounding non-vulval hypodermal cells.
(TIF)

S4 Fig Genetically separable functions of RGL-1. **A)** *let-60*(gf); *rgl-1(tm2255)* animals rescued by VPC-specific expression of GEF dead (R326E) RGL-1 fail to respond to *ral-1*-directed vs. control GFP RNAi. **B)** *let-60*(gf); *rgl-1(tm2255)* animals rescued by VPC-specific expression of wild-type RGL-1 rescue responsiveness to *ral-1*-directed but not control GFP RNAi. **C)** The *lin-31(n301)* reduced function (rf) mutation bypassed the putative 1°-promoting but not the putative 2°-promoting activity of *rgl-1*, revealed by the *tm2255* mutation enhancing ectopic 1° induction. **D)** *reIs10*[P_*lin-31*_::*ral-1(Q75L)* + P_*myo-2*_::*gfp*] suppressed the level of ectopic 1° induction in *let-60(n1046*gf*)*, as previously described for *reEx24* [10]. **E)** Repeat of the bypass experiment in Fig 4F with new strains that were re-derived from previously used strains. *rgl-1(gk274305)* (nonsense) further suppressed ectopic 1° induction, while *rgl-1(gk274304)* (putative GEF dead) failed to further suppress ectopic 1° fate induction, consistent with a GEF-independent 1°-promoting activity of RGL-1. The first two columns (*n1046*gf and *reIs10[ral-1(act)]*; *n1046*gf) were re-derived from crossing into the original *reIs10[ral-1(act)]*; *n1046*gf double mutant and re-isolating both genotypes. *reIs10[ral-1(act)]*; *n1046*gf; *rgl-1(gk274304)* and *reIs10[ral-1(act)]*; *n1046*gf; *rgl-1(gk274305)* were generated by crossing *gk274304* and *gk274305* hemizygous males to *reIs10[ral-1(act)]*; *n1046*gf; *rgl-1(tm2255)* and tracking *tm2255* by PCR to obtain *gk274304* and *gk274305* homozygotes. All four genotypes were scored concurrently from freshly isolated animals generated at the same time (animals were not passaged in culture prior to scoring). Y axis represents mean ectopic 1° cells, white labels number of animals counted, error bars show S.E.M. P value calculated via Mann-Whitney test.
(TIF)

S5 Fig RGL-1 interacts genetically with the 1°-promoting AGE-1/PI3K-PDK-1-AKT-1 cascade. *akt-1(mg144*gf*)* enhances 1° induction in *let-60(n1046*gf*)* animals, and this enhancement is blocked by **A)** *rgl-1(tm2255)* and **B)** *rgl-1(ok1921)*, though not to the original baseline. The *lin-45(n2506)* under-induced mutant is partially suppressed by **C)** *pdk-1(mg142*gf*)* not **D)** *akt-1(mg144*gf*)*. Unlike other assays, data shown are total induced VPCs, not ectopic 1°s. *daf-18(ok480)* enhances 1° induction in *let-60(n1046*gf*)* animals, and this enhancement is blocked by the **E)** *rgl-1(gk275305)* nonsense mutation but not the **F)** *rgl-1(gk275304*) R361Q putative GEF dead mutation. **G)** *jip-1*-directed RNAi suppressed the increase in ectopic 1° induction in the *let-60(n1046*gf*)* background conferred by *daf-18(ok480*), compared to *gfp(RNAi)*. **H)** *pdk-1*- and *rgl-1*-directed RNAi similarly suppress *daf-18(ok480*) *let-60(n1046*gf*)* ectopic 1° induction. **I)** The *jip-1* deletion mutation, *jip-1(tm6137)*, enhances *let-60(n1046*gf*)* alone but suppresses *daf-18(ok480*) *let-60(n1046*gf*)*, consistent with JIP-1 performing two functions. **J)** The observed genetic interactions are consistent with RGL-1 and JIP-1 functioning in the AGE-1-PDK-1-AKT-1 1° promoting cascade, as described for mammalian orthologs (Tian *et al*., 2002; Hao Wong and Feig, 2008). Except for *lin-45(*rf*)*, Y axis represents mean ectopic 1° cells, white labels number of animals counted, error bars show S.E.M. P value calculated via Mann-Whitney test or ANOVA.
(TIF)

S6 Fig PCR detection of *rgl-1(ok1921)*. **A)** A scale schematic of the *rgl-1* gene, the *ok1921* lesion, and detection primers. **B)** Agarose gel of +/+, *ok1921*/+ and *ok1921/ok1921* single animal PCR reactions.
(TIF)

S7 Fig PCR detection of *rgl-1(tm2255)*. **A)** A scale schematic of the *rgl-1* gene, the *tm2255* lesion, and detection primers. **B)** Agarose gel of +/+, *tm2255*/+ and *tm2255*/*tm2255* single animal PCR reactions.
(TIF)

S1 Table Strains. This table lists all strains used in this study, comprehensive genotype, and specific figures in which the reagents are used. “Results” indicates that the reagent is used broadly throughout the study, and the reader should refer to the Results.
(DOCX)

S2 Table Primers. A list of all primers used in this study.
(DOCX)
