# Supplementary figures and images for: Developmental fidelity is imposed by genetically separable RalGEF activities that mediate opposing signals
Source: PLoS Genet. 2019 May 14;15(5):e1008056. doi: 10.1371/journal.pgen.1008056 (PMC6534338; doi:10.1371/journal.pgen.1008056)

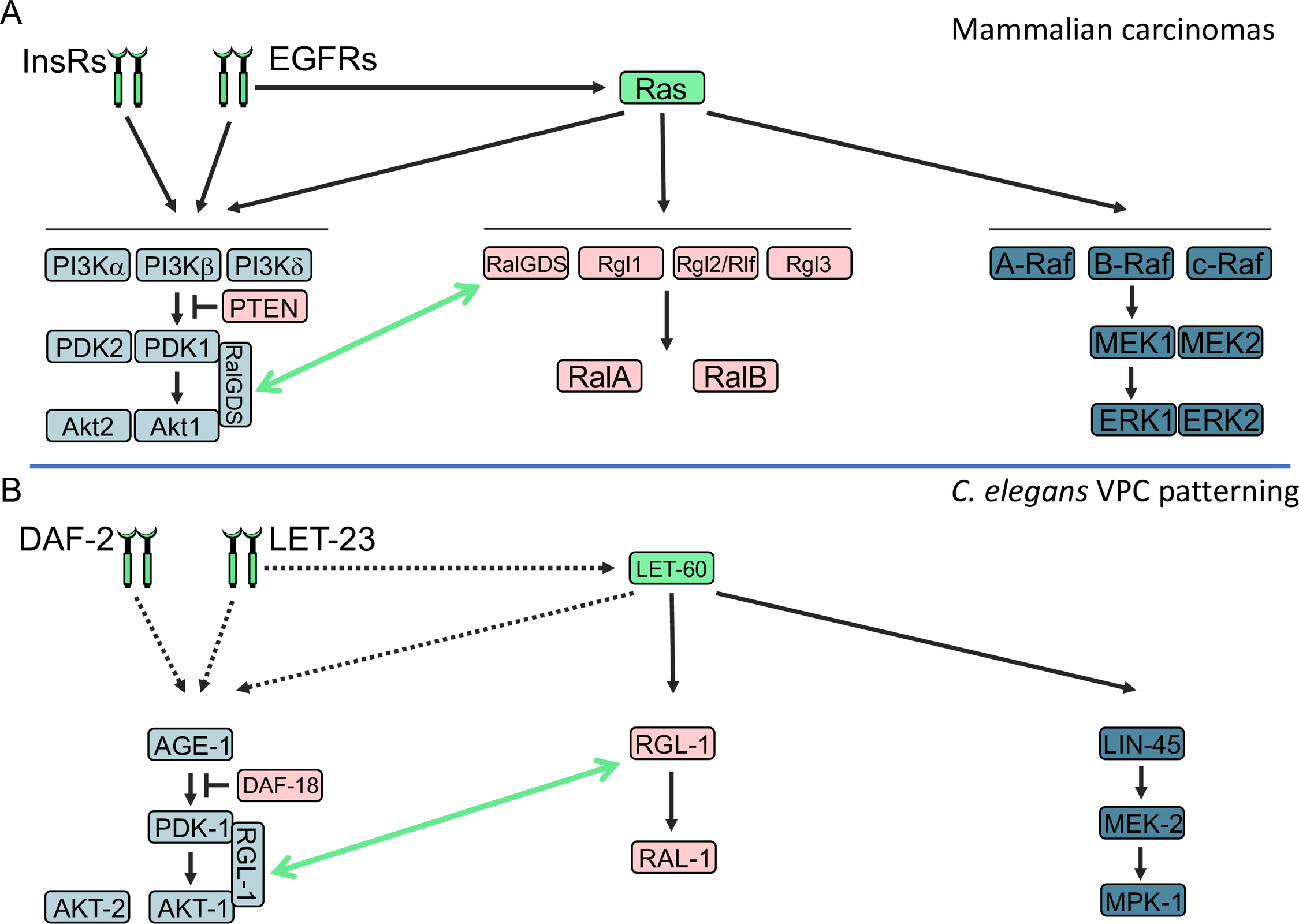

Supplement: S1 Fig — Signaling relationships in A) Mammalian carcinomas and B) C. elegans VPC fate patterning. Historically, mammalian interactions have been shown directly, while C. elegans interactions were deduced from a combination of phenotypes, genetic epistasis, and inferences from biochemical relationships among mammalian orthologs. Color coding is the same as in other figures: blue = 1°-promoting, rose = 2°-promoter, dark = necessary and sufficient signal, light = modulatory signal. Green = both 1°- and 2°-promoting (rather than green, RGL-1 is shown in two places, with a green two-headed arrow denoting possible dual function in both non-canonical 1°-promoting and canonical 2°-promoting roles). Activation of C. elegans AGE-1/PI3K by a receptor other than DAF-2, or by LET-60/Ras, has not been suggested in the literature, and hence is indicated by dotted lines. The interactions between RGL-1, PDK-1 and AKT-1 are inferred from genetic relationships in this study, and have not been shown directly. JIP-1, a potential intermediary between Akt and RalGDS/RalGEFs inferred from mammalian biochemical analyses in the Feig lab, is not shown. (TIF) [file pgen.1008056.s001.tif]

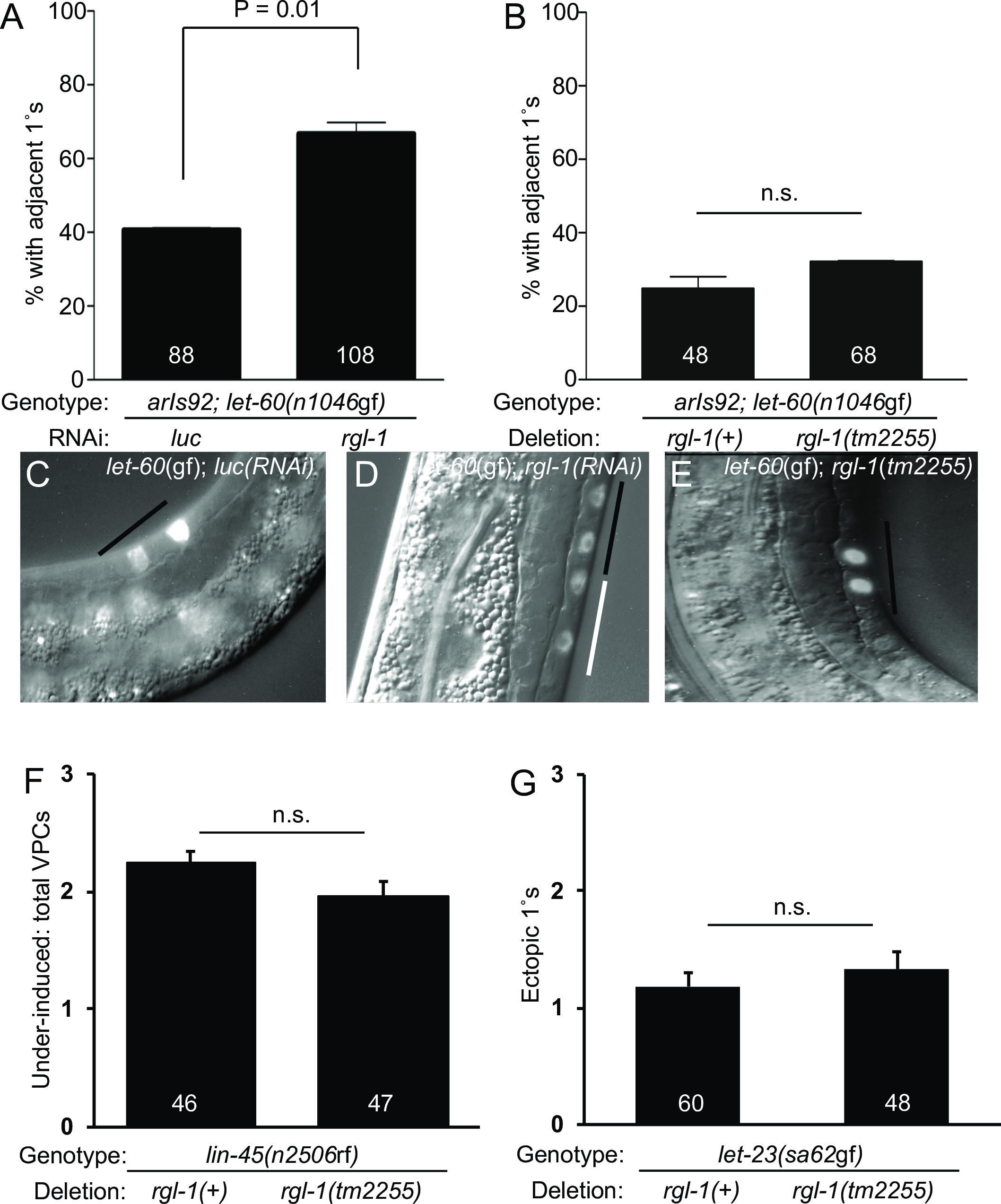

Supplement: S2 Fig — A) Percent Pn.px-staged let-60(gf) L3 larvae with CFP-positive lineages neighboring the P6.p lineage (P5.p or P7.p derived) with luc(RNAi) vs. rgl-1(RNAi). Shown are average percentages of animals with adjacent 1° cell fate. B) Percent Pn.px-staged let-60(gf) L3 larvae with CFP-positive lineages neighboring the P6.p lineage (P5.p or P7.p derived) with rgl-1(+) or rgl-1(tm2255Δ). Y axis is percent adjacent 1°s, white numbers in bars are number of animals scored per genotype. C-E) Expression of the 1° fate reporter arIs92 Pegl-17∷cfp-lacZ in VPC daughters. Overlaid DIC and CFP fluorescence images of C) let-60(n1046gf); luc(RNAi), D) let-60(n1046gf); rgl-1(RNAi) and E) let-60(n1046gf); rgl-1(tm2255Δ) at the Pn.px stage. The black bar indicates P6.px and white bar indicates P7.px cells. F) Hypo-induced lin-45(n2506) background with and without tm2255. Y axis is total induced VPCs (0 = vulvaless, 3 = normal wild-type vulva.) White numbers are number of animals assayed. G) let-23(sa62gf) with and without tm2255. Y axis is mean ectopic 1° induction. Error bars show S.E.M. P value calculated via Mann-Whitney test. (TIF) [file pgen.1008056.s002.tif]

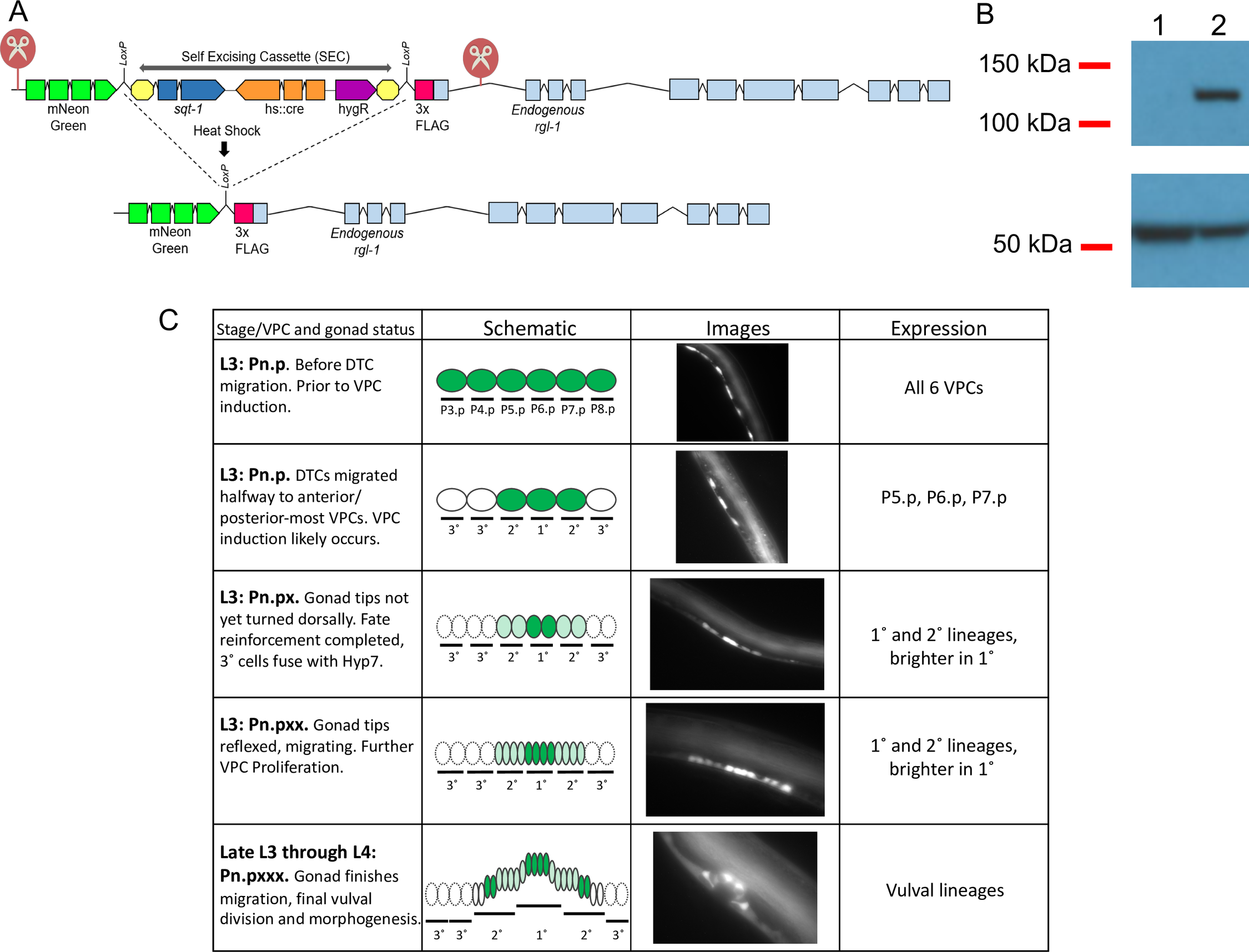

Supplement: S3 Fig — A) SEC positive-negative selection strategy for tagging rgl-1 with CRISPR. B) Western blot validation of the rgl-1 tag before (left) and after (right) SEC excision. The expected band of 129 kD (RGL-1+mNG+3xFlag) was observed. 55 kD α-tubulin loading control is shown below. C) We used a combination of DIC analysis of VPCs and migration of the gonadal distal cells for staging to characterize the dynamic pattern of GFP expression from sEx14985 [51] over time. Initial expression in naïve VPCs is uniform. Around the time of induction, expression is restricted to presumptive vulval lineages. Later expression, after the first cell division, remains higher in 1° than 2° linages. Later stages show low levels of expression in surrounding non-vulval hypodermal cells. (TIF) [file pgen.1008056.s003.tif]

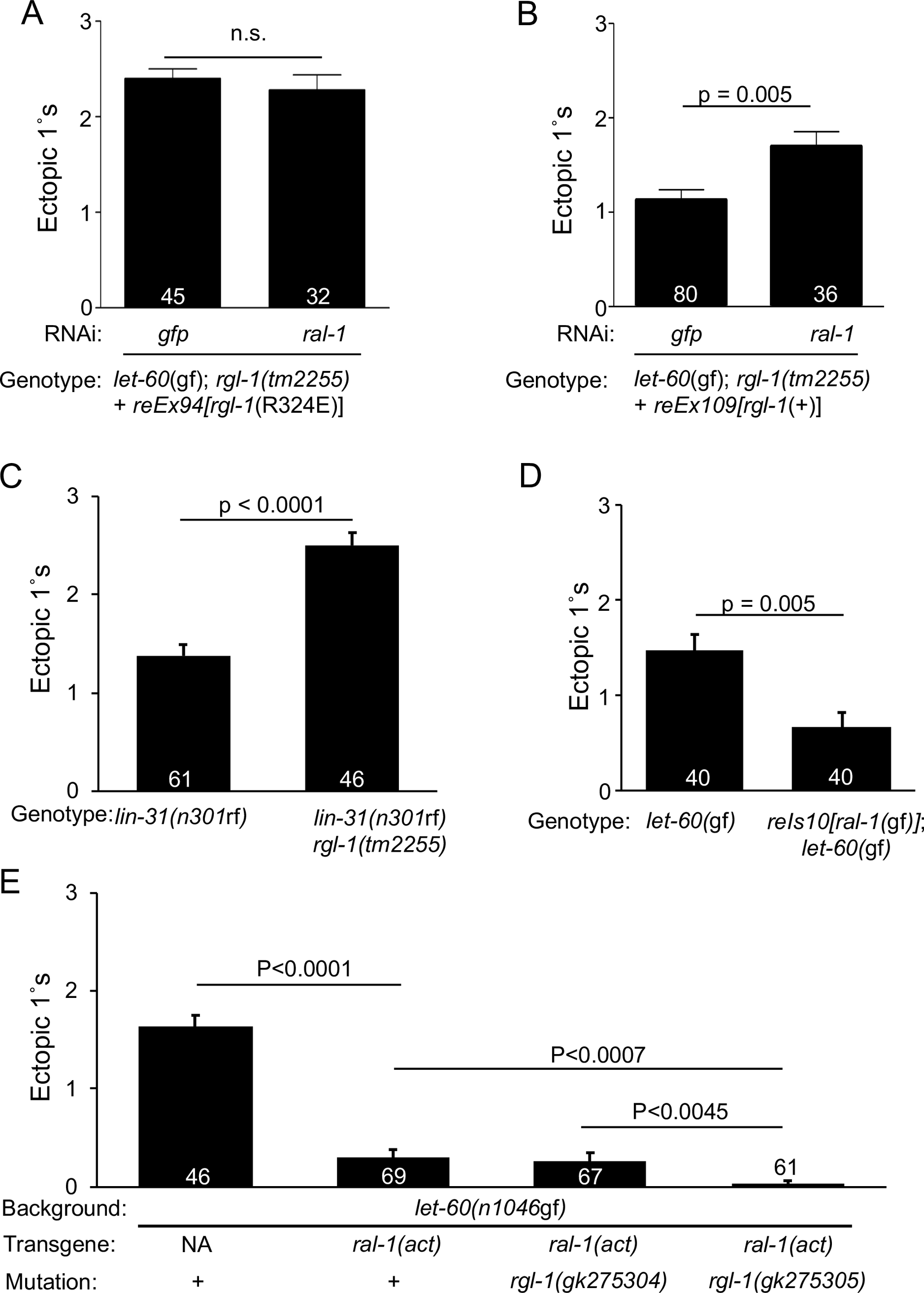

Supplement: S4 Fig — A) let-60(gf); rgl-1(tm2255) animals rescued by VPC-specific expression of GEF dead (R326E) RGL-1 fail to respond to ral-1-directed vs. control GFP RNAi. B) let-60(gf); rgl-1(tm2255) animals rescued by VPC-specific expression of wild-type RGL-1 rescue responsiveness to ral-1-directed but not control GFP RNAi. C) The lin-31(n301) reduced function (rf) mutation bypassed the putative 1°-promoting but not the putative 2°-promoting activity of rgl-1, revealed by the tm2255 mutation enhancing ectopic 1° induction. D) reIs10[Plin-31::ral-1(Q75L) + Pmyo-2::gfp] suppressed the level of ectopic 1° induction in let-60(n1046gf), as previously described for reEx24 [10]. E) Repeat of the bypass experiment in Fig 4F with new strains that were re-derived from previously used strains. rgl-1(gk274305) (nonsense) further suppressed ectopic 1° induction, while rgl-1(gk274304) (putative GEF dead) failed to further suppress ectopic 1° fate induction, consistent with a GEF-independent 1°-promoting activity of RGL-1. The first two columns (n1046gf and reIs10[ral-1(act)]; n1046gf) were re-derived from crossing into the original reIs10[ral-1(act)]; n1046gf double mutant and re-isolating both genotypes. reIs10[ral-1(act)]; n1046gf; rgl-1(gk274304) and reIs10[ral-1(act)]; n1046gf; rgl-1(gk274305) were generated by crossing gk274304 and gk274305 hemizygous males to reIs10[ral-1(act)]; n1046gf; rgl-1(tm2255) and tracking tm2255 by PCR to obtain gk274304 and gk274305 homozygotes. All four genotypes were scored concurrently from freshly isolated animals generated at the same time (animals were not passaged in culture prior to scoring). Y axis represents mean ectopic 1° cells, white labels number of animals counted, error bars show S.E.M. P value calculated via Mann-Whitney test. (TIF) [file pgen.1008056.s004.tif]

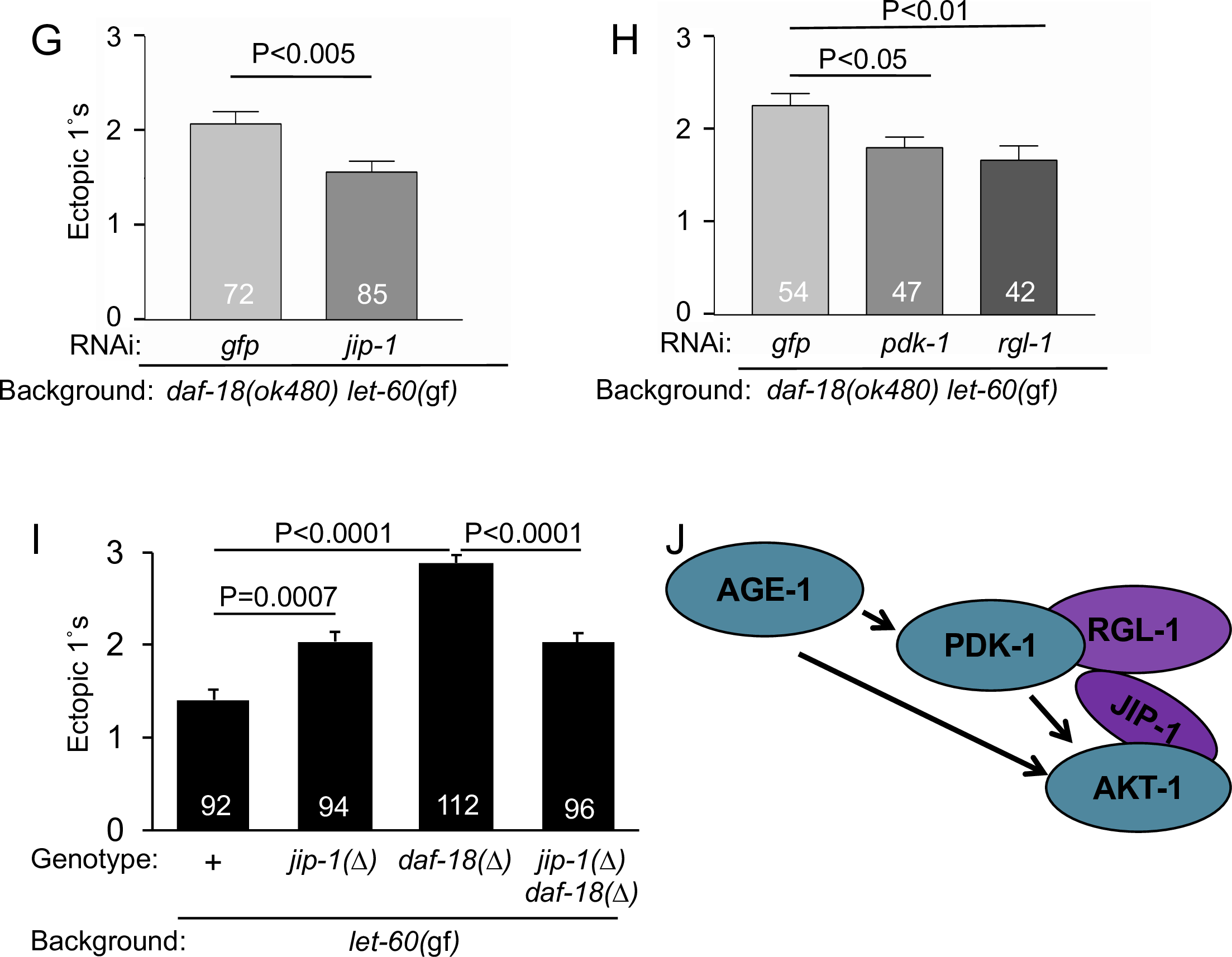

Supplement: S5 Fig — akt-1(mg144gf) enhances 1° induction in let-60(n1046gf) animals, and this enhancement is blocked by A) rgl-1(tm2255) and B) rgl-1(ok1921), though not to the original baseline. The lin-45(n2506) under-induced mutant is partially suppressed by C) pdk-1(mg142gf) not D) akt-1(mg144gf). Unlike other assays, data shown are total induced VPCs, not ectopic 1°s. daf-18(ok480) enhances 1° induction in let-60(n1046gf) animals, and this enhancement is blocked by the E) rgl-1(gk275305) nonsense mutation but not the F) rgl-1(gk275304) R361Q putative GEF dead mutation. G) jip-1-directed RNAi suppressed the increase in ectopic 1° induction in the let-60(n1046gf) background conferred by daf-18(ok480), compared to gfp(RNAi). H) pdk-1- and rgl-1-directed RNAi similarly suppress daf-18(ok480) let-60(n1046gf) ectopic 1° induction. I) The jip-1 deletion mutation, jip-1(tm6137), enhances let-60(n1046gf) alone but suppresses daf-18(ok480) let-60(n1046gf), consistent with JIP-1 performing two functions. J) The observed genetic interactions are consistent with RGL-1 and JIP-1 functioning in the AGE-1-PDK-1-AKT-1 1° promoting cascade, as described for mammalian orthologs (Tian et al., 2002; Hao Wong and Feig, 2008). Except for lin-45(rf), Y axis represents mean ectopic 1° cells, white labels number of animals counted, error bars show S.E.M. P value calculated via Mann-Whitney test or ANOVA. (TIF) [file pgen.1008056.s005.tif]

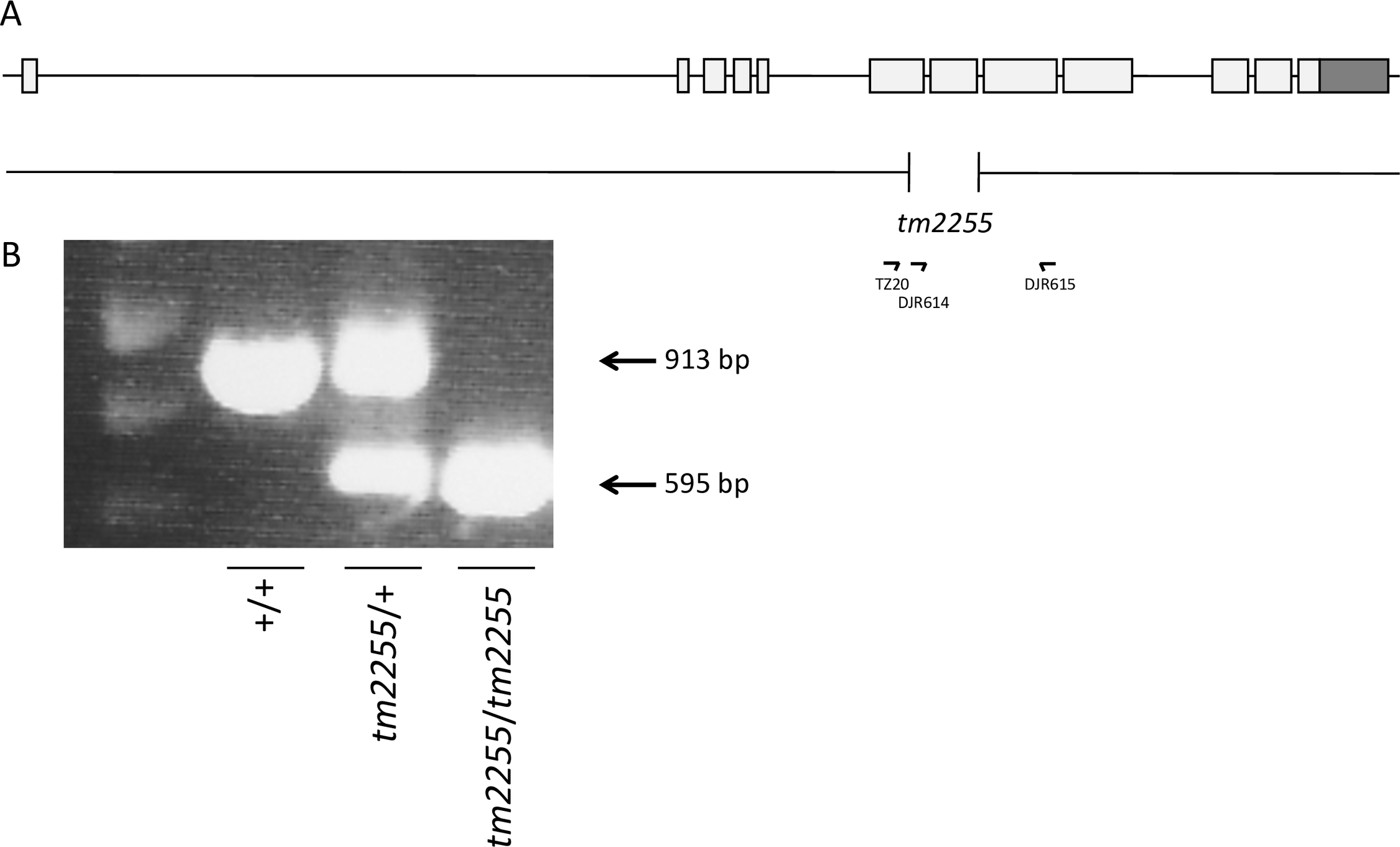

Supplement: S6 Fig — A) A scale schematic of the rgl-1 gene, the ok1921 lesion, and detection primers. B) Agarose gel of +/+, ok1921/+ and ok1921/ok1921 single animal PCR reactions. (TIF) [file pgen.1008056.s006.tif]

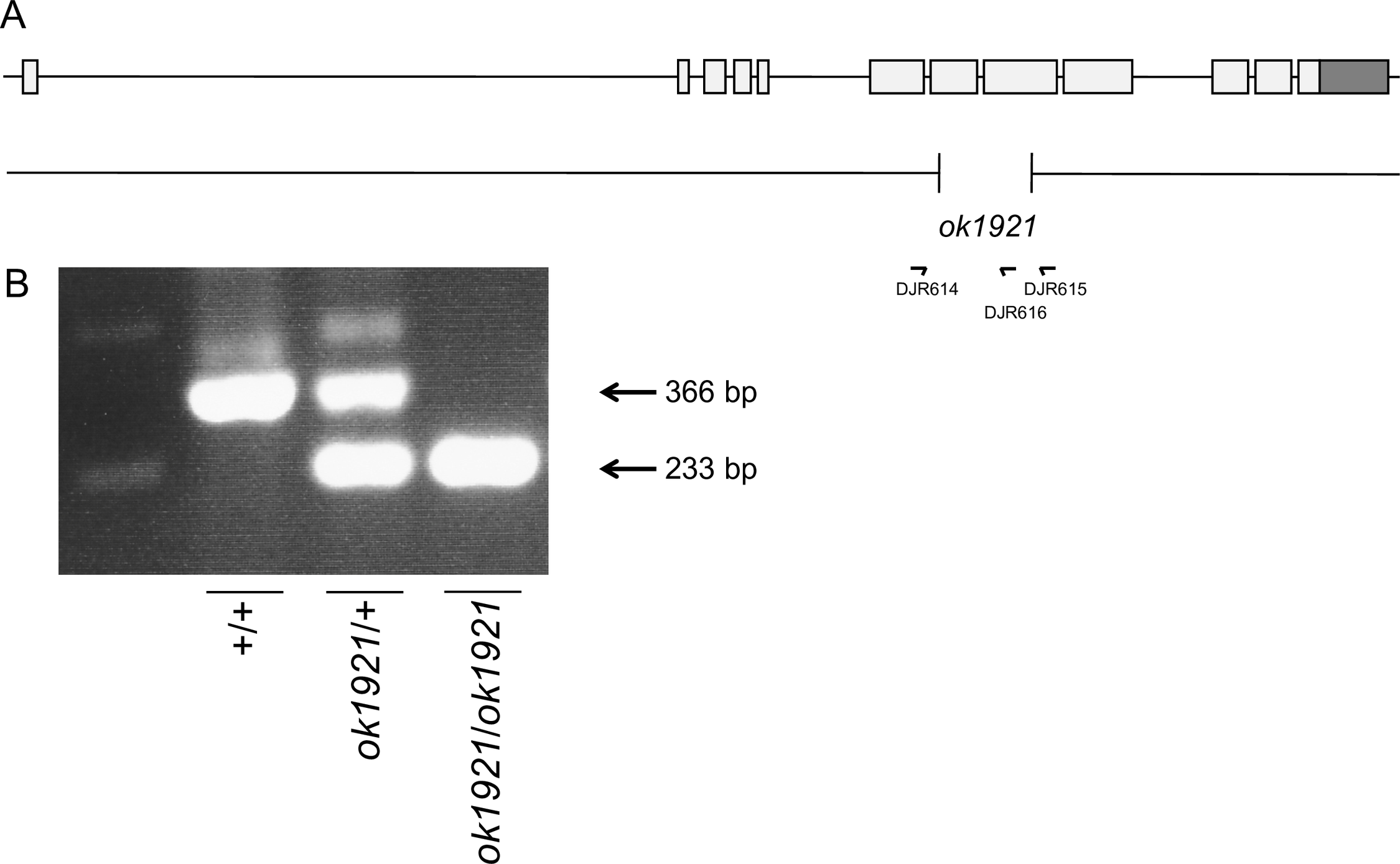

Supplement: S7 Fig — A) A scale schematic of the rgl-1 gene, the tm2255 lesion, and detection primers. B) Agarose gel of +/+, tm2255/+ and tm2255/tm2255 single animal PCR reactions. (TIF) [file pgen.1008056.s007.tif]
