# Supplementary material for: Developmental fidelity is imposed by genetically separable RalGEF activities that mediate opposing signals
Source: PLoS Genet. 2019 May 14;15(5):e1008056. doi: 10.1371/journal.pgen.1008056 (PMC6534338; doi:10.1371/journal.pgen.1008056)
Supplement: S1 Table — This table lists all strains used in this study, comprehensive genotype, and specific figures in which the reagents are used. “Results” indicates that the reagent is used broadly throughout the study, and the reader should refer to the Results. (DOCX) [file pgen.1008056.s008.docx]

**Table S1 - Strains**

| **Strain** | **Genotype** | **Application** |
| --- | --- | --- |
| DV3311 | *ral-1(tm2760)* / *qC1 [dpy-19(e1259*ts*) glp-1(q339) nIs189[P_myo-2_::gfp)]]* | Inspecting mutant VPCs |
| DV2924 | *ral-1(tm5205)* / *qC1 [dpy-19(e1259*ts*) glp-1(q339) nIs189[P_myo-2_::gfp]]* | Inspecting mutant VPCs |
| MT2124 | *let-60(n1046*gf*)* IV | Fig. 2B,C,E, baselines throughout |
| DV2214 | *let-60(n1046*gf*)* IV (2x outcrossed) | Results |
| DV2215 | *let-60(n1046*gf*)* IV (2x outcrossed) | Results |
| RB1576 | *rgl-1(ok1921)* X | Starting reagent |
| DV2194 | *rgl-1(ok1921)* X (4x outcrossed) | throughout |
| FX2255 | *rgl-1(tm2255)* X | Starting reagent |
| DV2175 | *rgl-1(tm2255)* X (4x outcrossed) | throughout |
| DV2937 | *ral-1(tm5205)* III / *qC1 [dpy-19(e1259*ts*) glp-1(q339) nIs189[P_myo-2_::gfp]]*; *let-60(n1046*gf*)* IV | Fig. 2F |
| DV2190 | *let-60(n1046*gf*)* IV; *rgl-1(tm2255)* X | Fig. 2D,F,G |
| DV2248 | *let-60(n1046*gf*)* IV; *rgl-1(ok1921)* X | Fig. 2F,G |
| DV2251 | *let-60(n1046*gf*)* IV; *lon-2(e678)* X | Fig. 2G |
| DV2252 | *let-60(n1046*gf*)* IV; *fax-1(gm27)* *lon-2(e678)* X | Fig. 2G |
| DV2191 | *let-60(1046*gf*)* IV; *arIs92[Pegl-17::NLS-cfp::LacZ* + *Pttx-3::gfp]* V | Fig. S2A, C, D |
| DV2750 | *let-60(n1046*gf*)* IV; *arIs92[Pegl-17::NLS-cfp::LacZ* + *Pttx-3::gfp]* V; *rgl-1(tm2255)* | Fig. S2B, E |
| WU49 | *lin-45(n2506*rf*) unc-24(e138)* IV | Fig. S2F, S5E, S5F |
| DV2783 | *lin-45(n2506rf)* *unc-24(e138)* IV; *rgl-1(tm2255)* X | Fig. S2F |
| PS1524 | *unc-4(e120) let-23(sa62*gf*)* II | Fig. S2G |
| DV2958 | *unc-4(e120) let-23(sa62*gf*)* II; *rgl-1(tm2255)* X | Fig. S2G |
| BC14985 | *dpy-5(e907)* I; *sEx14985[rCesF28B4.2::gfp* + *PCeh361(dpy-5(*+*))]* | Fig. 3 |
| DV3312 | *rgl-1(re179[mNeonGreen^3xFlag::rgl-1])* X | Fig. S3 |
| VC40420 | *ral-1(gk628801*rf*)* III (unoutcrossed) | Results |
| DV2942 | *ral-1(gk628801*rf*)* III (6x outcrossed) | Results |
| DV2799 | *ral-1(gk628801)* III; *let-60(n1046*gf*)* IV | Fig. 4C |
| VC20011 | *rgl-1(gk275304)* X (unoutcrossed) | Results |
| DV2883 | *rgl-1(gk275304)* X (4x outcrossed) | Results |
| VC40052 | *rgl-1(gk275305)* X (unoutcrossed) | Results |
| DV2884 | *rgl-1(gk275305)* X (4x outcrossed) | Results |
| DV2764 | *let-60(n1046*gf*)* IV; *rgl-1(gk275304)* X | Fig. 4D |
| DV2765 | *let-60(n1046*gf*)* IV; *rgl-1(gk275305)* X | Fig. 4D |
| DV2537 | *let-60(n1046*gf*)* IV; *rgl-1(tm2255)* X; *reEx94*[*P_lin-31_::rgl-1(R324E)* + *P_myo-2_::GFP*] | Fig. 4E, S4A |
| DV2538 | *let-60(n1046*gf*)* IV; *rgl-1(tm2255)* X; *reEx95*[*P_lin-31_::rgl-1(R324E)* + *P_myo-2_::GFP*] | Fig. 4E, S4A |
| DV2736 | *let-60(n1046*gf*)* IV; *rgl-1(tm2255)* X; *reEx109*[*P_lin-31_::rgl-1(*+*)* + *P_myo-2_::GFP*] | Fig. 4E, S4B |
| DV2737 | *let-60(n1046*gf*)* IV; *rgl-1(tm2255)* X; *reEx110*[*P_lin-31_::rgl-1(*+*)* + *P_myo-2_::GFP*] | Fig. 4E, S4B |
| MT301 | *lin-31(n301)* II | Fig. S4C |
| DV2763 | *lin-31(n301)* II; *rgl-1(tm2255)* | Fig. S4C |
| DV2140 | *let-60(n1046*gf*)* IV; *reEx24[P_lin-31_::ral-1(Q75L), P_myo-2_::gfp)]* | Zand, 2011, derived *reIs10* |
| DV2335 | *reIs10*[*P_lin-31_::ral-1(Q75L)* + *Pmyo-2::GFP*] I | Results |
| DV2699 | *reIs10*[*P_lin-31_::ral-1(Q75L)* + *Pmyo-2::GFP*] I; *let-60(n1046*gf*)* IV | Fig. 4F, S4D |
| DV2700 | *reIs10*[*P_lin-31_::ral-1(Q75L)* + *Pmyo-2::GFP*] I; *let-60(n1046*gf*)* IV; *rgl-1(tm2255)* X | Fig. 4F |
| DV3643 | *let-60(n1046*gf*)* IV | Fig. S4E |
| DV3644 | *reIs10*[*P_lin-31_::ral-1(Q75L)* + *Pmyo-2::GFP*] I; *let-60(n1046*gf*)* IV | Fig. S4E |
| DV3645 | *reIs10*[*P_lin-31_::ral-1(Q75L)* + *Pmyo-2::GFP*] I; *let-60(n1046*gf*)* IV; *rgl-1(gk275304)* X | Fig. S4E |
| DV3646 | *reIs10*[*P_lin-31_::ral-1(Q75L)* + *Pmyo-2::GFP*] I; *let-60(n1046*gf*)* IV; *rgl-1(gk275305)* X | Fig. S4E |
| CB2065 | *dpy-11(e224) unc-76(e905)* | *akt-1* balancer |
| GR1310 | *akt-1(mg144*gf*)* V | Fig. 5 |
| DV2746 | *let-60(n1046*gf*)* IV; *akt-1(mg144*gf*)* V | Fig. 5A, S5A |
| DV2747 | *let-60(n1046*gf*)* IV; *akt-1(mg144*gf*)* V; *rgl-1(tm2255)* X | Fig. 5A, S5A |
| GR1318 | *pdk-1(mg142*gf*)* X | Fig. 5 |
| DV2773 | *let-60(n1046*gf*)* IV; *pdk-1(mg142*gf*)* X | Fig. 5B, S5B |
| DV2782 | *let-60(n1046*gf*)* IV; *pdk-1(mg142*gf*)* X | Fig. 5B |
| DV2844 | *let-60(n1046*gf*)* IV; *pdk-1(mg142*gf*)* X | Fig. 5B |
| SP934 | *unc-1(e538) dpy-3(e27)* X | Fig. 5B |
| DV2774 | *unc-1(e538) dpy-3(e27)* *rgl-1(tm2255)* X | Fig. 5B |
| DV2819 | *pdk-1(mg142*gf*) rgl-1(tm2255)* X | Fig. 5B |
| DV2822 | *let-60(n1046*gf*)* IV; *pdk-1(mg142*gf*) rgl-1(tm2255)* X | Fig. 5B |
| DV2791 | *lin-45(n2506rf)* *unc-24(e138)* IV; *akt-1(mg144*gf*)* V | Fig. S5F |
| DV2795 | *lin-45(n2506rf)* *unc-24(e138)* IV; *pdk-1(mg142*gf*)* X | Fig. S5E |
| RB712 | *daf-18(ok480) IV* | Starting reagent |
| DV2855 | *daf-18(ok480)* 2x outcrossed | Fig. 5 |
| DV2510 | *daf-18(ok480)* *let-60(n1046*gf*)* IV | Fig. 5C, D, S5C, S5D, S5G, S5H |
| DV2535 | *daf-18(ok480)* *let-60(n1046*gf*)* IV; *rgl-1(tm2255)* X | Fig. 5C |
| DV2509 | *daf-18(ok480)* *let-60(n1046*gf*)* IV; *rgl-1(tm2255)* X | Fig. 5D |
| DV2679 | *jip-1(km18)* II | Fig. S5I |
| DV3013 | *jip-1(km18)* II; *let-60(n1046*gf*)* IV | Fig. S5I |
| DV3358 | *jip-1(tm6137)* II (4x outcrossed) | Fig. S5I |
| DV3505 | *jip-1(tm6137)* II; *let-60(n1046*gf*)* IV | Fig. S5I |
| DV3506 | *jip-1(tm6137)* II; *daf-18(ok480) let-60(n1046*gf*)* IV | Fig. S5I |
| DV2696 | *rgl-1(ok1921)* 8x outcrossed | Fig. 6 |
| DV2697 | *rgl-1(tm2255)* 8x outcrossed | Fig. 6 |
