# Supplementary material for: Developmental fidelity is imposed by genetically separable RalGEF activities that mediate opposing signals
Source: PLoS Genet. 2019 May 14;15(5):e1008056. doi: 10.1371/journal.pgen.1008056 (PMC6534338; doi:10.1371/journal.pgen.1008056)
Supplement: S2 Table — A list of all primers used in this study. (DOCX) [file pgen.1008056.s009.docx]

**Table S2 – Primers**

| Name | Sequence | Use |
| --- | --- | --- |
| DJR614 | 5’-GAGCAACTGACGTTTTGGGATGC-3’ | *rgl-1(ok1921)* genotyping |
| DJR615 | 5’-GATCTGGAGTGGAGTGCATTGG-3’ | *rgl-1(ok1921)* genotyping |
| DJR616 | 5’-CGAAAAGCTCCCCACTTCGACG-3’ | *rgl-1(ok1921)* genotyping |
| DJR778 | 5’-tagacaatttaggcccaaaacccccg-3’ | *ral-1(gk628801)* genotyping |
| DJR779 | 5’-Ccaaattttcagcctaaaatctcttcccaatacc-3’ | *ral-1(gk628801)* genotyping |
| RGL-1F | 5’-gcgggatccgaaaaaATGGCTACGCGTTACTGGGGTGACG-3’ | Cloning *rgl-1a* cDNA |
| RGL-1R | 5’-ataagaatgcggccgctTTACAAGTAGCCACTGCTCCATG-3’ | Cloning *rgl-1a* cDNA |
| DRC1 | 5’-AGATGCTAGCTGACGGAGATGTGGG-3’ | *pdk-1(mg142*gf*)* genotyping |
| DRC2 | 5’-AAATGTGGCTGGAATGTAGGCGTGC-3’ | *pdk-1(mg142*gf*)* genotyping |
| FSM4 | 5’-ATATTCGAGGAGTCGGTGGTCC-3’ | *daf-18(ok480)* genotyping |
| FSM5 | 5’-GAGGCTACCGGATAATGTGC-3’ | *daf-18(ok480)* genotyping |
| FSM6 | 5’-GGCAACGAATGAATACGCAGG-3’ | *daf-18(ok480)* genotyping |
| FSM7 | 5’-GAAGTCAAGCCGCTCTTCC-3’ | *rgl-1(tm2255)* genotyping |
| FSM8 | 5’-GGAGAACTGCTGGAGAACG-3’ | *rgl-1(tm2255)* genotyping |
| FSM9 | 5’-CCGTTCCCTGACATTCGG-3’ | *rgl-1(tm2255)* genotyping |
| HS160 | 5’-ACACCTTCGTATCCTTGTGGGTTTAAGAGCTATGCTGGAAACAG-3’ | *rgl-1* sgRNA Cas-9 plasmid #1 |
| HS161 | 5’-GGTCTGAGTTCTTCTGACGAGTTTAAGAGCTATGCTGGAAACAG-3’ | sgRNA Cas-9 plasmid #2 |
| DJR769 | 5’-CACCTCCTATTGCGAGATGTCTTG-3’ | Universal sgRNA plasmid Mutagenesis |
| HS120 | 5’-ccagtcacgacgttgtaaaacgacggccagtcgccggcaccgcatccattcacagtgtac-3’ | Left homology arm |
| HS159 | 5’-AGGGAGGCCATGTTGTCCTCCTCTCCCTTGGAGACCATACGCGTAGCCATttaagattggtctgagttcttctgacg-3’ | Left homology arm |
| HS122 | 5’-CGACGACAAGCGTGATTACAAGGATGACGATGACAAGAGAGGATCTGGAATGGCTACGCGTTACTGGG-3’ | Right homology arm |
| HS123 | 5’- taacaatttcacacaggaaacagctatgaccatgttatcgaagtggcactcagcttcatc-3’ | Right homology arm |
| HS125 | 5’-CTTGTCACTGTAAGGGAAGATTTCC3’- | *rgl-1* CRISPR genotyping |
| HS126 | 5’-TTGTCCTCCTCTCCCTTGG-3’ | *rgl-1* CRISPR genotyping |
| HS127 | 5’ ACGTAGAATGTTCCAGAGTTCCAG-3 | *rgl-1* CRISPR genotyping |
| KM1 | 5’-GCCGGCCCGAATACGAAGCGAAGATAATCAGCAAGTGGATCG-3’ | R324E mutagenesis |
| KM2 | 5’-GATCCACTTGCTGATTATCTTCGCTTCGTATTCGGGCCGG-3’ | R324E mutagenesis |
